# Translational insights and clinical challenges of targeting cancer stem cells

**DOI:** 10.1038/s41392-026-02887-y

**Published:** 2026-08-13

**Authors:** Mehreen Ahmed, Amr Al-Haidari, Shruti Agarwal, Jianmin Sun, Emma U. Hammarlund, Lars Rönnstrand, Kenneth J. Pienta, Özge Tatli, Julhash U. Kazi

**Affiliations:** 1 Division of Translational Cancer Research, Department of Laboratory Medicine, Lund University, Lund, Sweden; 2 Lund Stem Cell Center, Department of Laboratory Medicine, Lund University, Lund, Sweden; 3 Lund University Cancer Centre (LUCC), Lund University, Lund, Sweden; 4 Department of Urology, University of Rochester Medical Center, Rochester, NY USA; 5 Institute of Medical Genetics and Genomics, Sir Ganga Ram Hospital, New Delhi, India; 6 School of Basic Medical Sciences, Ningxia Medical University, Yinchuan, China; 7 Tissue Development and Evolution (TiDE), Department of Experimental Medical Science, Lund University, Lund, Sweden; 8 Division of Hematology, Oncology and Radiation Physics, Skåne University Hospital, Lund, Sweden; 9 The Cancer Ecology Center, Brady Urological Institute, Johns Hopkins School of Medicine, Baltimore, MD USA; 10 Department of Molecular Biology and Genetics, Faculty of Engineering and Natural Sciences, Istanbul Medeniyet University, Istanbul, Türkiye; 11 Present Address: Department of Surgery and Cancer, Imperial College London, London, UK

**Keywords:** Cancer stem cells, Cancer microenvironment

## Abstract

Cancer stem cells (CSCs) are tumor cell subsets with self-renewal, multilineage differentiation, and tumor-initiating capacity that sustain cancer initiation, progression, metastasis, and relapse. Targeting CSCs therefore represents a promising route to improve the durability of cancer treatment. However, translation of this approach into routine care has been slow because of the biological complexity and clinical constraints. This review discusses current concepts of CSC origin and plasticity, the criteria used to define CSCs across different tumor types, and the marker systems as well as high-resolution technologies that are used to track CSC states. Developmental pathways, growth factor and cytokine cascades, as well as microenvironmental and stress responses that control CSC maintenance and therapy resistance are explored with a focus on their tractability as drug targets. We then discuss mechanisms through which CSCs escape chemotherapy, radiotherapy, and targeted agents. We review current efforts to use these pathways in designing small molecules, antibodies, cellular therapies, and vaccines aimed at CSC compartments. Heterogeneity within and between tumors, dynamic interconversion between CSC and non-CSC states, and support from specialized niches are considered as major barriers for clinical trial design, biomarker development, and response assessment. Emerging single-cell, spatial, and lineage tracing approaches, together with organoid and ex vivo platforms, are reviewed as tools that can bridge preclinical models and patient samples and guide the development of CSC-directed combination regimens. The goal is to outline translational principles that can guide future strategies for integrating CSC-focused interventions with established therapies to improve long-term disease control.

## Introduction

Cancer stem cells (CSCs) are of considerable interest in contemporary cancer biology because they provide a mechanistic link between intratumoral heterogeneity, tumor propagation, therapeutic resistance, and disease recurrence.^1,2^ CSCs are commonly defined as tumor-cell subsets with the capacity for self-renewal, multilineage differentiation, and tumor initiation. These properties parallel key aspects of normal stem-cell biology, while being shaped by oncogenic alterations and malignant microenvironments.^3,4^ The CSC concept emerged from functional transplantation studies in hematologic malignancies and was subsequently extended to solid tumors.^5–7^ These studies showed that tumor cells can be organized hierarchically, analogous to normal tissues in which stem cells occupy the apex of the cellular hierarchy. Within this framework, only a subset of tumor cells possesses extensive tumor-initiating and self-renewal capacities, thereby contributing to intratumoral heterogeneity and sustained tumor growth.^1^

Current understanding of CSC biology has moved beyond a simple hierarchical model.^1^ In some cancers, tumor growth follows a hierarchical organization in which transformed stem or progenitor cells reside near the apex and generate more differentiated progeny. In others, differentiated or partially differentiated tumor cells can reacquire stem-like properties through cellular plasticity.^2,8^ These models are regarded as complementary rather than mutually exclusive. Dedifferentiation can be promoted by epithelial-to-mesenchymal transition-like transcriptional programs, epigenetic remodeling, hypoxia, inflammatory cytokines, cytotoxic therapy, and disruption of the local niche.^9,10^ Therefore, rigorous CSC identification requires the integration of marker-based enrichment with functional assays, serial propagation or limiting-dilution transplantation, lineage history, and state-resolved molecular profiling. Recent advances in single-cell transcriptomics, spatial profiling, lineage tracing, genetic barcoding, and organoid platforms have transformed the field by resolving rare therapy-tolerant states, mapping CSC-associated programs within intact niches, and linking pre-existing or therapy-induced cell states to relapse.^10,11^

CSCs have important implications for cancer therapy. Accumulating evidence suggests that CSCs exhibit greater resistance to conventional treatments, including chemotherapy and radiotherapy, than the bulk tumor-cell population.^10,12^ This resistance has been attributed to several mechanisms, including enhanced DNA repair, activation of pro-survival signaling pathways, and the ability to enter quiescent or slow-cycling states. Because many anticancer therapies preferentially target rapidly proliferating cells, quiescent CSCs may survive treatment and subsequently regenerate the tumor, contributing to disease relapse, metastasis, and poor clinical outcomes.^1,10^ Clinically, interest in CSCs is driven by their capacity to withstand standard and targeted therapies through quiescence or dormancy, enhanced DNA damage checkpoints and repair, low basal reactive oxygen species and antioxidant buffering, ALDH-mediated detoxification, ABC transporter-mediated drug efflux, suppression of apoptosis, ferroptosis defense, autophagy, mitochondrial quality control, and protection by hypoxic, stromal, immune, and extracellular-matrix niches.^10,12^ These survival programs do not operate in isolation but are coordinated by signaling networks that overlap with normal development and tissue repair. Such pathway interdependence helps explain why CSCs can adapt when a single node is inhibited and why resistant disease often reflects both selection of pre-existing clones and therapy-induced phenotypic switching. It also highlights a translational challenge that CSC-directed interventions should aim to reduce malignant stemness without irreversibly damaging normal stem and progenitor compartments relying on related pathways for tissue maintenance.

The therapeutic status of CSC targeting therefore remains promising but clinically unresolved. Preclinical studies support strategies that inhibit stemness pathways, block niche-derived survival signals, disrupt metabolic or redox defenses, promote differentiation, induce ferroptosis or apoptosis, and combine CSC-directed approaches with chemotherapy, radiotherapy, targeted therapy, or immunotherapy.^10,12^ Clinically, agents directed against signaling molecules, ALDH-associated metabolism, telomerase, CSC-associated antigens, and CSC-enriched immune targets have entered early-phase or selected later-phase studies.^13,14^ However, responses have generally been variable and are often limited by toxicity, pathway redundancy, heterogeneous biomarker expression, inadequate pharmacodynamic confirmation of CSC depletion, and the absence of validated CSC-specific endpoints.^10,14^ The focus of this review is to synthesize these advances from a translational perspective. We discuss how CSCs arise and are identified, how high-resolution technologies refine the CSC concept, how developmental, stress-response, growth-factor, cytokine, metabolic, and niche-associated pathways sustain CSC behavior, and how these mechanisms contribute to therapy resistance. Finally, we evaluate emerging therapeutic strategies and clinical challenges, with the goal of defining principles for rational combination regimens and biomarker-guided trial designs that can integrate CSC-directed interventions with established cancer therapies to improve durable disease control.

## Origin and identification of cancer stem cells

The origin of CSCs remains under active investigation, and there is ongoing debate regarding how CSCs should be operationally defined and identified. Current models propose that CSCs arise either from transformation of normal stem cells or from dedifferentiation of more mature cancer cells. These concepts frame the high-resolution approaches discussed below and their impact on defining CSC state, lineage, function, and niche.

### Conceptual models of origin

The conceptualization of CSC models has evolved markedly over the past decades, moving from static hierarchical views to dynamic, adaptive, and systems-level frameworks that collectively describe tumor heterogeneity, evolution, and therapy resistance. CSC theory traces its lineage from 19th-century embryonal rest hypotheses through mid-20th-century transplantation experiments.^15^ Later, two primary models, the hierarchical and plasticity models, have provided the foundation for understanding CSC biology. More recent extensions, including mimicry, metabolic, and systems-integration models, have further refined this landscape by emphasizing CSC adaptability, environmental interactions, and regulatory complexity.

The traditional hierarchical model proposes that tumors are organized as hierarchies sustained by a rare, self-renewing CSC population at the apex. In this framework, CSCs originate from normal tissue stem cells or committed progenitors that acquire oncogenic mutations and subsequently generate differentiated cancer cells through largely unidirectional differentiation. This model is supported by studies in AML demonstrating that only CD34^+^CD38^-^ cells could initiate leukemia in immunodeficient mice through serial transplantation, whereas more differentiated populations lacked this capacity.^5,6^ Similar hierarchical organization has been described in solid tumors, including breast cancer, showing that CD44^+^CD24^-/low^ cells displayed markedly enhanced tumor-initiating capacity compared to non-CSC populations.^7^

Hierarchical organization explains intratumoral heterogeneity through asymmetric and symmetric CSC divisions, generating coexisting cell states with distinct proliferative capacities, phenotypes, and therapeutic sensitivities.^16–18^ Experimental lineage-tracing studies have provided important in vivo support for this model by demonstrating that defined normal stem-cell populations can serve as cells of origin for cancer. For example, inducible Cre-based lineage tracing has shown that LGR5^+^ intestinal crypt stem cells can initiate intestinal tumors following acquisition of oncogenic mutations, while more differentiated or committed intestinal populations typically require additional context and are less efficient under homeostatic conditions.^19–22^ However, lineage-tracing evidence predominantly identifies the cell type in which transformation is initiated rather than conclusively defining the cellular sources that sustain long-term CSC activity during tumor progression. Several studies have highlighted technical and conceptual limitations of lineage tracing, including promoter specificity, incomplete or artifactual labeling, and the inability of many systems to capture stress-induced phenotypic transitions.^22–25^ Moreover, genetic barcoding and clonal evolution analyses in glioblastoma and squamous cell carcinoma have shown dynamic clonal architectures that are difficult to reconcile with a fixed, unidirectional hierarchy in all settings, suggesting that stem-like tumor-propagating capacity may be more dynamic than a single immutable lineage.^26–28^

The plasticity or dynamic CSC model challenges the rigid hierarchical view by proposing that stemness is a reversible cellular state rather than a fixed lineage property.^10,29–31^ In this framework, differentiated cancer cells can reacquire stem-like characteristics through dedifferentiation driven by microenvironmental cues, therapeutic stress, or activation of developmental signaling pathways. This bidirectional interconversion is often associated with epithelial-to-mesenchymal transition (EMT)-like transcriptional programs and chromatin remodeling.^9,32–36^ Supporting evidence includes observations that non-CSC breast cancer cells can spontaneously convert to CSC-like states through ZEB1 activation, that radiation or chemotherapy induces stem-cell marker expression and tumor-initiating capacity in glioblastoma, and that stromal-derived factors such as hepatocyte growth factor (HGF) can restore stemness in differentiated colorectal cancer cells.^10,35–42^ Despite this functional evidence, the extent to which dedifferentiation contributes to CSC maintenance in vivo remains actively debated. Many studies supporting plasticity rely on marker expression, in vitro assays, or transplantation into permissive hosts, which may overestimate the frequency or stability of dedifferentiated states. In contrast, several inducible lineage-tracing experiments in skin, intestine, and lung cancers suggest that differentiated tumor cells contribute less to long-term tumor propagation during unperturbed growth in some models, but can contribute substantially after tissue injury or therapeutic perturbation.^22,43–45^ These findings suggest that dedifferentiation is highly context-dependent rather than constitutive, functioning as an adaptive response to environmental or treatment-induced stress rather than a default mechanism of tumor growth.

Importantly, the apparent frequency and durability of dedifferentiated CSC-like states depend strongly on experimental context. In relatively unperturbed or homeostatic tumor growth, lineage-tracing and clonal analyses in epithelial tumors often show that long-term propagation is biased toward pre-existing stem/progenitor-like compartments or stable clonal lineages, with a more limited contribution from fully differentiated tumor cells.^22,43,44^ By contrast, plasticity becomes more evident when tissue architecture or niche constraints are disrupted, such as after tissue injury, niche depletion, inflammation, hypoxia, transplantation stress, radiotherapy, chemotherapy, or targeted therapy.^10,37–42,44,46–48^ These conditions can activate regenerative wound-healing programs, EMT-like transcriptional states, HIF-dependent stress responses, WNT/Notch/TGF-β pathways, NF-κB/STAT3 inflammatory signaling, and chromatin remodeling programs that lower barriers to state transition.^10,35–42,47,48^ Thus, dedifferentiation should be interpreted as a conditional adaptive response favored by damage, stress, or niche reorganization, rather than as a default or uniformly stable mechanism of tumor maintenance. This distinction is important because in vitro sphere assays, marker-based sorting, tissue dissociation and xenotransplantation into permissive hosts can amplify stress-induced plasticity and may overestimate the prevalence or persistence of dedifferentiated CSC states in native, unperturbed tumors.^49–52^

Current understanding increasingly supports a unified framework in which hierarchical organization and phenotypic plasticity coexist rather than operate as mutually exclusive models.^53,54^ In this view, tumors may originate from defined stem or progenitor populations that establish an initial hierarchy, while differentiated cancer cells retain the capacity to re-enter stem-like states under selective stress responses such as therapy, hypoxia, or niche disruption. Recent lineage-tracing, genetic barcoding, and single-cell longitudinal studies support this dynamic hierarchy by demonstrating stable lineage contributions during tumor growth alongside episodic state switching following perturbation.^44,46,55–57^ Thus, the apparent contradiction between stem-cell origin and dedifferentiation reflects differences in temporal scale, experimental context, and selective stress response rather than fundamentally opposing biological mechanisms. CSC identity emerges as a probabilistic and environmentally regulated state superimposed upon a partially constrained lineage architecture.

Systems-level integration of signaling pathways, metabolic programs, and microenvironmental cues further shapes CSC stemness.^10,58^ Multi-omics studies show that CSCs are not locked into a single metabolic program, but instead display metabolic plasticity, dynamically switching among glycolysis, oxidative phosphorylation (OXPHOS), and lipid/alternative-fuel metabolism depending on oxygen levels, nutrient availability, and therapeutic or microenvironmental stress.^59–61^ For example, under nutrient-rich or proliferative conditions, CSCs may favor glycolysis, while under nutrient deprivation or therapeutic stress they may shift to OXPHOS or fatty acid oxidation to maintain stemness.^60,62^ This flexibility is supported by signaling networks involving developmental pathways that interface with metabolic enzyme expression and mitochondrial regulation.^63,64^ Moreover, CSCs do not operate in isolation; they engage in metabolic symbiosis with stromal and immune cells within the tumor microenvironment.^65,66^ For instance, lactate secreted by hypoxic bulk tumor cells may fuel oxidative metabolism in CSCs; whereas stromal fibroblasts or tumor-associated macrophages may secrete metabolites or cytokines that support CSC metabolism and survival.^61,65^ Spatial and single-cell transcriptomic and metabolomic data from glioma studies suggest that CSCs occupy distinct niches with unique metabolic states and that metabolic trajectories correlate with spatial positions such as tumor core vs periphery, and cell-cell interactions.^67–69^ These systems-level models integrate classical stem-cell-like signaling with metabolic reprogramming and spatial heterogeneity. They suggest that targeting CSCs effectively will require not only inhibition of signaling pathways but also interventions targeting metabolic flexibility and the microenvironmental support networks that enable CSC survival.

### Unique properties distinguishing CSCs from other cancer cells

CSCs possess several defining properties that distinguish them from the bulk of tumor cells. While many of these traits are rooted in the hierarchical, plastic, and systems-level frameworks outlined above, their functional consequences such as therapy resistance, enhanced invasiveness, immune evasion, and dormancy, collectively make CSCs a uniquely resilient tumor subpopulation.^70^ As introduced above, CSCs exhibit enhanced DNA repair, activation of checkpoint pathways, and expression of drug efflux transporters.^71^ These intrinsic mechanisms allow CSCs to withstand cytotoxic and targeted therapies more effectively than non-stem tumor cells. Additionally, many CSCs reside in quiescent or slow-cycling states, a property central to the hierarchical model, which enables passive evasion of therapies targeting highly proliferative cells.

The plasticity and EMT programs as described in origin models also underpin the elevated invasive potential of CSCs. Many CSC-like populations upregulate matrix metalloproteinases (MMPs), facilitating extracellular matrix degradation and invasion, and frequently engage EMT-associated transcriptional circuits that promote motility and apoptosis resistance.^32,72–76^ These features can position CSC-like cells as contributors to metastatic initiation, consistent with their capacity to transition between epithelial, mesenchymal, and hybrid phenotypic states under microenvironmental pressures. In parallel, CSCs display an enhanced ability to evade immune surveillance, a phenomenon intertwined with the mimicry and systems-level models described earlier. CSCs can downregulate antigen-presentation machinery, increase immune checkpoint expression, and secrete immunosuppressive cytokines, thereby shaping a tumor microenvironment that suppresses effector immune responses and promotes regulatory T-cell (Treg) expansion.^77–79^ These strategies allow CSCs to persist despite immune-mediated pressure and may reduce the effectiveness of immunotherapies.

**Fig. 1:**
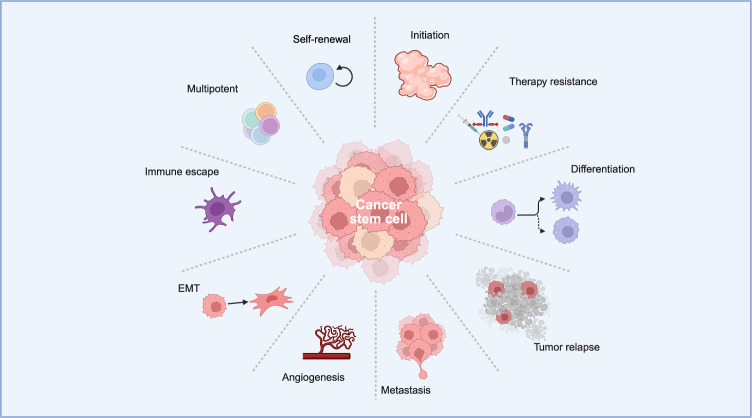
Characteristics and roles of CSCs in therapy resistance. CSCs display self-renewal, tumor-propagating capacity, differentiation potential, therapy resistance, EMT-associated invasion, immune escape, angiogenesis, metastasis and relapse-associated properties. CSCs can generate heterogeneous tumor-cell progeny, survive radiotherapy and drug treatments, interact with niche components and contribute to recurrence and metastatic spread in selected contexts. Created in BioRender. Uddin, K. (2026) https://BioRender.com/32o86nl

A further distinguishing feature of CSCs is their capacity to enter and maintain dormancy. Dormant CSCs can survive prolonged periods of nutrient deprivation, hypoxia, and therapeutic stress by adopting low-proliferative, metabolically restrained states.^80,81^ Their eventual reactivation can drive tumor recurrence and metastatic outgrowth, sometimes years after apparently successful initial treatment, posing a major obstacle to durable clinical remission, suggesting the need for therapeutic strategies that specifically target quiescent CSC populations and prevent their reawakening.^82,83^ The interplay among therapy resistance, invasiveness, immune evasion, and dormancy creates a resilient CSC compartment that is a contributor to therapeutic failure and relapse (Fig. 1).

### Identification and markers of CSCs

The identification of CSCs relies on a combination of functional assays, phenotypic enrichment strategies, and molecular profiling, as no single marker or technique can definitively define CSC identity across tumor types. Herein, in vivo functional assays remain the gold standard. Orthotopic limiting-dilution xenotransplantation, followed by serial transplantation, provides direct evidence of tumor-propagating capacity and long-term self-renewal under microenvironmental conditions that most closely resemble the native tumor niche.^49^

Several functional enrichment tools can prospectively isolate CSC-like states prior to in vivo validation. High aldehyde dehydrogenase (ALDH) activity detected using the ALDEFLUOR assay enriches for tumor-initiating cells in many cancers, reflecting metabolic programs associated with stemness.^84^ Hoechst 33342 dye-efflux-based side-population assays capture cells with elevated ABC transporter activity, a feature linked to drug resistance and often enriched in CSC compartments.^85^ Reporter constructs for developmental pathways such as WNT, Notch, or Hedgehog have also been used to track stemness-associated transcriptional activity in vivo.^86^ These approaches facilitate rapid enrichment for stem-like populations, which can subsequently be assessed using sphere assays or limiting-dilution transplantation; however, ALDH^high^, dye-efflux, and sphere-forming phenotypes also occur in normal or stress-induced states, indicating that functional readouts alone are insufficiently specific.^50^

Phenotypic marker-based enrichment provides a practical route for isolating CSC-enriched fractions, yet no surface antigen functions as a universal CSC marker. The interpretation of any given CSC marker is tumor and assay-dependent and often depends on expression level and splice isoform. Primary limiting-dilution xenograft studies identified tumor-initiating fractions defined by marker combinations in specific cancers, for example CD44^high^CD24^low^ in breast cancer,^7^ CD44^+^CD24^+^EpCAM^+^ in pancreatic cancer,^87^ CD45^−^CD90^+^ in hepatocellular carcinoma,^88^ and CD133^+^ fractions in glioblastoma and colorectal cancer.^89–91^ However, subsequent studies also show that these markers can be broadly expressed, dynamically regulated, or non-exclusive for tumor-propagating activity. For instance, CD133 is widely expressed in primary colonic epithelium and does not uniquely define tumor-initiating cells in metastatic colorectal cancer, where both EpCAM^+^CD133^+^ and EpCAM^+^CD133^−^ subsets can serially propagate tumors.^92^ Likewise, CD44 is broadly detectable in normal and transformed colorectal epithelium.^93^ These observations suggest that CSC enrichment requires explicit reporting of gating strategy together with isoform identity and expression level.

EpCAM^high^CD44^+^ populations, often refined with CD166, are enriched in tumor-initiating cells in colorectal cancer,^94^ whereas metastatic competence has been linked more specifically to CD44 variant isoforms, particularly CD44v6, which marks clonogenic CSCs and supports migration and metastatic outgrowth.^95,96^ In gastric cancer, antibodies recognizing the CD44v9 epitope identify subsets associated with recurrence,^97^ consistent with functional evidence that CD44 variant isoforms can promote stress resistance via xCT-dependent redox control.^98^ Conversely, EpCAM, originally described as a colorectal carcinoma antigen,^99^ is broadly expressed across carcinomas and metastases,^100^ and is therefore best viewed as an epithelial/tumor gating marker rather than a CSC marker per se, unless combined with additional, tumor-contextual markers.^94^ Finally, several proposed markers are tumor-specific rather than tumor-restricted. For example, ITGA7 has been reported as a functional CSC surface marker in esophageal squamous cell carcinoma^101^ and has also been independently validated as a functional, targetable marker of glioblastoma stem-like cells.^102^ Collectively, CSC markers should be treated as tumor-context-specific enrichment tools that require reporting with isoform and expression threshold detail, and validation through functional assays including serial transplantation or limiting dilution alongside state-resolved profiling.

A major challenge in CSC identification is the intrinsic plasticity of tumor cells. Dissociation, sorting, therapeutic exposure, or microenvironmental shifts can rapidly induce transitions into or out of stem-like states, altering both functional behaviors and marker expression. To resolve these transitions, contemporary approaches incorporate single-cell transcriptomics, lineage tracing, and spatially resolved profiling to map stemness signatures, lineage trajectories, and niche-dependent behaviors with greater precision.^44,103–105^

### High-resolution technologies in CSC research

Recent advances in scRNA-seq, spatial transcriptomics (ST)/high-plex imaging, lineage tracing, and patient-derived organoids have reframed CSC biology. Rather than viewing CSCs as fixed, marker-defined subpopulations, these approaches support a model in which stemness often emerges as a dynamic and reversible cell state shaped by transcriptional programs, epigenetic priming, clonal selection, and spatially defined microenvironmental niches. High-resolution profiling has been particularly influential in three areas: (i) resolving heterogeneous malignant states and rare therapy-tolerant programs within the same tumor, (ii) locating stem-like programs within intact tissue architectures, such as invasive fronts, hypoxic layers, perivascular regions, and (iii) establishing causal links between CSC-like states, clonal behavior, treatment selection, and relapse through time-resolved lineage reconstruction. Together, these technologies move CSC research from enrichment and correlation toward state-resolved, niche-aware, and longitudinally validated models of tumor maintenance and resistance.

#### Single-cell transcriptomes

scRNA-seq has reshaped CSC concepts by showing that many tumors contain a continuum of malignant transcriptional states, including progenitor-like/stem-like programs and stress-adapted or mesenchymal-like programs that can expand under treatment. Early studies in glioblastoma and melanoma demonstrated pronounced intratumoral diversity and the coexistence of multiple malignant cell states within individual tumors, providing a foundation for interpreting CSCs as state(s) within a heterogeneous ecosystem rather than a single stable compartment.^106,107^ In head-and-neck cancer, single-cell profiling similarly revealed distinct malignant programs and highlighted partial EMT/stress-associated programs linked to invasion and microenvironmental interactions, reinforcing the idea that stem-like traits can couple to migratory or therapy-adaptive states.^108^

Beyond cell type catalogs, trajectory-oriented analyses, like pseudotime and RNA velocity, are used to infer state transitions between more stem-like and more differentiated phenotypes and to identify intermediate states consistent with CSC plasticity.^109,110^ In practice, these methods have helped convert CSC biology from a static hierarchy into a dynamic landscape in which cells can move between phenotypes depending on therapy and niche signals, providing mechanistic hypotheses for relapse and minimal residual disease. Single-cell profiling has also improved how CSC-associated programs are interpreted in genetically complex tumors. Inferring large-scale copy-number alterations from scRNA-seq data helps separate malignant from non-malignant cells and links cell states to subclonal architecture, enabling the question “which subclones preferentially occupy stem-like programs?” to be addressed in the same dataset.^106^ State scoring and regulatory-network inference tools, such as GSVA/AUCell-like gene program scoring and SCENIC-based network inference, have further enabled quantitative assessment of pathway activity and regulatory circuits associated with stemness, stress tolerance, and partial EMT at single-cell resolution.^111^

A major conceptual advance has come from single-cell multi-omics, which better connects CSC states to regulatory mechanisms. CITE-seq links transcriptomes to surface-protein phenotypes, helping reconcile CSC marker behavior with underlying programs.^112^ Joint RNA-chromatin accessibility approaches and chromatin potential frameworks show that regulatory changes can precede or predict transcriptional shifts, supporting a model in which CSC plasticity can be epigenetically primed before a full transcriptional transition is evident.^113,114^ This mechanistic layer is central for CSC biology because it provides plausible explanations for how tumors can rapidly regenerate stem-like programs after therapy or microenvironmental perturbation.

Technical caveats remain important but are best viewed as guardrails rather than limitations of the biology. Dissociation can induce stress-associated signatures, and batch effects or doublets can distort rare-state inference. Improved dissociation protocols, single-nucleus approaches, and careful computational QC reduce these risks.^51,52,115–118^ Ultimately, scRNA-seq-derived CSC states are most convincing when paired with functional validation, transplantation capacity or longitudinal evidence from lineage tracing.^119^

#### Spatial transcriptomics and high-plex imaging

Spatial technologies have had a distinct impact on CSC biology by reintroducing tissue architecture into models of stemness. While scRNA-seq reveals cell states, ST and in situ imaging show where those states occur and which microenvironmental features accompany them and are key for CSC survival and therapy tolerance.^120,121^ In glioblastoma, integrative spatial profiling has revealed a multi-layered organization of malignant programs and microenvironmental structure, supporting the concept that stem-like or regenerative programs can be regionally enriched and shaped by local cues rather than uniformly distributed throughout the tumor mass.^122,123^

Sequencing-based ST platforms, like original ST, Visium-like array systems, and bead-based high-resolution methods such as Slide-seq, map transcriptome-wide programs across intact sections and can resolve gradients of differentiation, stress response, and immune exclusion across tumor architecture.^120,121^ Imaging-based methods, for instance, MERFISH/seqFISH-style approaches, provide high spatial resolution for targeted gene sets, enabling direct visualization of cell-cell neighborhoods and local pathway activation patterns that can define CSC-supportive niches.^124,125^ Complementing RNA-level maps, high-plex protein imaging (CODEX, IMC, MIBI-TOF) has been used to quantify niche composition and receptor/ligand organization, tumor-immune proximity, stromal patterns, and immune checkpoint landscapes that can sustain or restrict CSC-like states.^126–129^

Spatial work has also sharpened CSC interpretation in tissues with well-defined normal stem-cell niches.^122,123^ For example, direct visualization of short-range WNT gradients in the intestinal stem-cell niche illustrates how positional information controls normal stemness and provides a conceptual template for how tumors may re-create or hijack analogous gradients to maintain CSC-like behavior at specific locations such as invasive fronts.^130^ Collectively, spatial technologies convert CSC biology from “which cells are stem-like?” to “which cells are stem-like, where, and under which niche constraints?”

#### Lineage tracing and clonal barcoding

Lineage tracing and clonal barcoding have had major impact on the causal interpretation of CSCs by testing whether stem-like states truly drive tumor growth, persistence, and relapse over time. Clonal analyses in solid tumors established that tumors can follow different growth rules, some consistent with hierarchical CSC-like propagation, others closer to neutral competition, suggesting that the CSC model is not uniform across cancers.^22,43^

Critically, lineage-based approaches have provided direct evidence that therapy can select for or reveal restricted populations with regenerative capacity. In glioblastoma, lineage-related studies demonstrated that a restricted cell population can propagate tumor regrowth after chemotherapy, supporting the concept that relapse can be seeded by cells that survive treatment and subsequently reacquire or maintain tumor-propagating potential.^131^ In AML, barcoding and longitudinal tracking showed that chemotherapy can enrich for leukemic-regenerating activity and that treatment regimens can shift which populations seed recurrence, reframing resistance as a problem of time-resolved state/clonal selection rather than baseline marker expression alone.^132,133^

High-complexity barcoding in PDX and other models has further shown that rare pre-existing clones can expand under therapy and that metastatic/relapse lesions can reflect distinct clonal contributions, emphasizing that CSC behavior is often embedded in clonal dynamics rather than being explained purely by cross-sectional marker status.^56,134^ Newer lineage recorders like CRISPR-based synthetic recorders and recombinase systems enable lineage reconstruction with single-cell readouts, accelerating efforts to connect fate history with transcriptional/epigenetic state.^135–137^ In human samples, naturally occurring mitochondrial mutations captured in single-cell datasets can serve as intrinsic lineage marks, enabling retrospective lineage inference without experimental labeling.^138,139^ These approaches place CSC biology into a temporal framework, identifying which states persist, which are induced, and which are transient intermediates under treatment, while also highlighting their limitations, such as barcode dropout, unequal labeling, and recorder dynamics, that require careful experimental design and computational correction.^140–143^

#### Organoids and ex vivo systems

Organoid systems have substantially advanced CSC biology by providing an experimentally tractable bridge between state discovery (single-cell/spatial profiling) and functional testing. Patient-derived tumor organoids (PDOs) preserve key features of tumor hierarchy and heterogeneity, allowing CSC-like programs to be interrogated under controlled conditions and across treatment exposures. In gastrointestinal cancers, organoid drug-response profiles have been shown to correlate with clinical outcomes in matched patients, supporting the use of PDOs for functional stratification and therapy prioritization.^144^

Organoids also support mechanistic dissection of how oncogenic lesions and microenvironmental constraints shape stemness. CRISPR engineering in intestinal organoids has been used to model stepwise colorectal tumorigenesis, creating controlled systems to test how combinations of driver alterations influence differentiation trajectories and stem-like programs.^145^ Importantly for CSC biology, organoids can be combined with single-cell profiling and perturbation workflows to test whether candidate CSC regulators identified in scRNA-seq or ST are required for persistence, regeneration, or therapy tolerance.

Co-culture formats extend organoids toward niche-aware CSC models. Air-liquid interface and immune co-culture systems preserve or reconstitute tumor-immune interactions, enabling analysis of immune pressure, immune evasion, and response to immunotherapy in a context where CSC-like states can be monitored longitudinally.^146,147^ These platforms are not complete microenvironments; vascular, myeloid, fibroblast, and biomechanical cues often require additional engineering, and culture conditions can bias clonal composition over time.^148^ Therefore, organoid findings are most powerful when integrated with spatial mapping and in vivo validation.

#### AI in CSC research

Modern machine learning tools increasingly extract biologically useful signals from single-cell and spatial datasets. Deep generative models and graph neural networks can identify stem-like and CSC-related gene programs, even in datasets affected by noise or batch effects.^149^ These approaches also support biomarker identification in clinical contexts by linking gene signatures to drug-response predictions that can guide treatment hypotheses.

After standard quality checks and normalization, batch-correction and integration merhods are typically applied as the next steps. Classical anchor-based integration and scalable embedding methods can handle differences between donors and technologies.^150,151^ Variational autoencoder frameworks such as scVI and scANVI integrate batch correction, denoising, uncertainty estimation, and cell state label transfer within a single probabilistic model.^149,152^ The scVI-tools ecosystem is particularly useful in these settings.^153,154^ Spatial data add another layer of complexity. Graph convolutional and graph attention models can combine gene expression with spatial proximity information.^155,156^ Heterogeneous graph learning extends this approach further by linking genes, cells, and tissue regions within a unified framework.^157^ This can help resolve fine-grained cell states and communication patterns. Once latent embeddings are learned, tools such as Uniform Manifold Approximation and Projection (UMAP) support visualization and trajectory mapping. Transfer learning can also allow researchers to map new patient data onto a reference atlas, or align single-cell and spatial measurements in order to locate rare cellular programs.^158,159^

Several studies show how these methods can translate CSC and stemness concepts into practical, testable signatures. In gastric cancer, one study combined single-cell sequencing with spatial and bulk transcriptomics, stemness has been scored in cancer cells using CytoTRACE, built co-expression modules, and screened candidate features through multiple machine learning approaches.^160^ A support vector machine based on five genes achieved strong classification performance for identifying high-stemness cells. Follow-up experiments pointed to JAK1/STAT3 signaling and showed increased sensitivity to common chemotherapies. In pancreatic cancer, stemness has been quantified using scRNA-seq data and used a community-detection approach on bulk RNA-seq data to identify co-occurring cell-state groups.^161^ One poor-prognosis community was enriched for aggressive basal-like cancer cells, specific macrophage subtypes, and myofibroblastic fibroblasts. This study also found a stemness continuum among cancer cells and fibroblasts that was associated with patient survival.^161^ A pan-cancer study constructed a stemness signature by analyzing 34 single-cell datasets and tested it across 10 immunotherapy cohorts.^162^ Higher stemness scores were associated with resistance to immune checkpoint therapy. A machine learning model based on this signature predicted immunotherapy response with moderate accuracy across validation and test sets. Taken together, these studies show that combining single-cell data with targeted machine learning approaches is starting to translate the concept of cancer stemness into clinically relevant, testable predictions, though much work remains to validate these tools across broader patient populations.

## Regulation of signaling pathways governing CSC behavior

The signaling networks that govern CSC behavior can be broadly divided into three levels of control: (i) developmental programs that guide lineage fate and are frequently reactivated in tumors; (ii) microenvironment- and stress-related processes that support survival during metabolic challenge, inflammation, or cytotoxic treatment; and (iii) cascades triggered by growth factors and cytokines that influence proliferation and adaptive responses. Together, these networks enable regulatory modules to interact and maintain CSC properties.

### Canonical CSC drivers - core developmental pathways

Developmental signaling routes often reappear in tumors and play a major part in preserving traits linked to CSCs. These systems guide fate choices during embryogenesis and, when reactivated, can support self-renewal, asymmetric division, and survival under therapeutic stress. Their activity also shapes transcriptional states that permit shifts between stem-like and differentiated phenotypes.

#### WNT/β-catenin signaling

WNT signaling is frequently deregulated in cancer and supports CSC phenotypes by sustaining self-renewal programs, promoting proliferation, and enabling treatment tolerance.^163–165^ The signaling comprises 19 secreted ligands and a large receptor/co-receptor repertoire, most commonly involving Frizzled (FZD) receptors together with LRP5/6, allowing context-dependent activation of canonical (β-catenin-dependent) and non-canonical (β-catenin-independent) branches.^163,164^

**Fig. 2:**
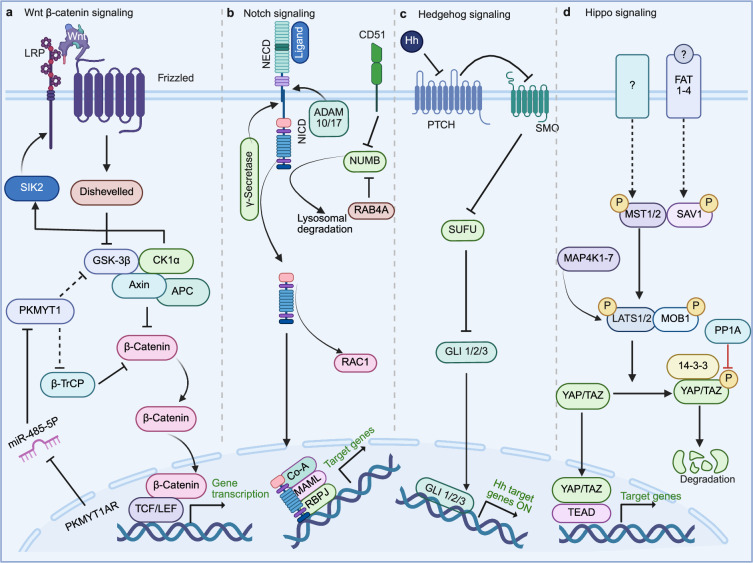
Overview of key CSC-associated developmental pathways: WNT/β-catenin, Notch, Hedgehog, and Hippo signaling. **a** WNT/β-catenin signaling begins when WNT ligands engage Frizzled receptors and LRP5/6 co-receptors, leading to stabilization of β-catenin and its nuclear interaction with TCF/LEF transcription factors. The lncRNA PKMYT1AR can sequester miR-485-5p, increasing PKMYT1 and supporting β-catenin stabilization by interfering with β-TrCP-mediated degradation and/or GSK-3β-dependent regulation. SIK2-mediated phosphorylation of LRP6 can further amplify pathway activity. **b** Notch signaling is initiated by ligand-dependent receptor activation followed by ADAM10/17 and γ-secretase cleavage, which releases the Notch intracellular domain (NICD). NICD translocates to the nucleus and cooperates with RBPJ/CSL and MAML to regulate transcription. **c** Hedgehog signaling is initiated when Hh ligands bind Patched (PTCH), relieving inhibition of SMO and promoting GLI transcription-factor activity. **d** The Hippo pathway is controlled by MST1/2 and MAP4K kinases that activate LATS1/2. Active LATS1/2 phosphorylate YAP and TAZ, causing cytoplasmic retention or degradation. When Hippo signaling is reduced, YAP and TAZ move into the nucleus and cooperate with TEAD transcription factors to promote genes involved in growth control and renewal capacity. Created in BioRender. Uddin, K. (2026) https://BioRender.com/3iaqueg

In the absence of WNT ligands, β-catenin is phosphorylated and targeted for degradation through a destruction complex containing AXIN, GSK-3β, APC, and CK1, which promotes ubiquitination of phosphorylated β-catenin through β-TrCP and proteasomal turnover (Fig. 2a). Disruption of this complex stabilizes β-catenin and enables nuclear translocation with induction of TCF/LEF target genes that reinforce stemness and survival.^163,164^ A clinically relevant genetic example is APC loss/truncation, which is common in colorectal cancer and compromises destruction-complex function, favoring β-catenin stabilization. In chemoresistant colorectal cancer (CRC), Sec62 was identified as an additional WNT-activating node that competitively disrupts APC-β-catenin binding, inhibits destruction-complex activity, and increases nuclear β-catenin transcriptional output.^166^ Functionally Sec62 expression reduces sensitivity to 5-FU and oxaliplatin, linking canonical β-catenin signaling to chemotherapy resistance through apoptosis suppression and CSC maintenance.^166^ A second canonical example comes from hepatocellular carcinoma, where liver CSC-enriched conditions identified FZD10 as a resistance-associated receptor, and elevated FZD10 expression promoted CSC traits and lenvatinib resistance via WNT/β-catenin and Hippo/YAP signaling.^167^

β-catenin-independent WNT signaling is typically triggered when WNT ligands engage FZD together with co-receptors such as ROR1/ROR2, or other context-specific partners such as RYK, activating downstream modules that reshape polarity, migration, cytoskeletal architecture, and transcriptional states linked to invasion and therapy persistence.^163–165^ In colon cancer cells, hypoxia altered the expression of non-canonical co-receptors ROR1/ROR2, supporting a transition from a more proliferative canonical WNT state toward a more migratory or aggressive non-canonical phenotype, illustrating how microenvironmental cues bias WNT branch usage.^168^ Importantly, non-canonical WNT outputs can also be context-dependent and tumor-suppressive. WNT5A-ROR2 signaling activated Hippo pathway components to suppress YAP1/TAZ activity and tumor growth in an experimental setting, suggesting that the biological consequence of non-canonical signaling depends on receptor context and downstream coupling.^169^ This contextual variability is consistent with clinical correlative observations linking WNT5A/ROR2 expression patterns to tumor differentiation states in hepatocellular carcinoma.^170^

CSC-associated WNT activity is also tuned by RNA-based regulation. In non-small cell lung cancer (NSCLC), the lncRNA PKMYT1AR promoted CSC maintenance by sustaining WNT signaling through a miRNA-mediated mechanism that protects β-catenin from degradation.^171^ In colorectal cancer, circ_0000467 promoted proliferation and stem-like features by activating TCF4/WNT/β-catenin output via miRNA sponging.^172^ Beyond RNA regulation, post-translational pathway modulation can also amplify WNT signaling. In multiple myeloma, oxidation of DVL3 promoted WNT/β-catenin activation and malignant phenotypes.^173^ Pathway crosstalk can further reinforce survival outputs. For example, in glioma, NF-κB1-linked transcriptional wiring intersected with WNT/β-catenin signaling to promote proliferation and suppress apoptosis.^174^

Epigenetic regulation refers to heritable and reversible control of gene expression that does not change DNA sequence, commonly mediated by chromatin modifiers that alter promoter/enhancer accessibility and transcriptional output. A CSC-relevant example is KDM1A in thyroid cancer, identified as an epigenetic modifier that maintained stemness via WNT/β-catenin signaling by repressing canonical WNT antagonists APC2 and DKK1.^175^ Functionally, pharmacologic KDM1A inhibition enhanced chemosensitivity, directly linking epigenetic reprogramming of WNT pathway regulators to treatment response.

In parallel to chromatin-based epigenetics, epitranscriptomic m^6^A RNA modification also altered effective pathway strength by increasing expression of WNT-relevant components such as FZD10 in liver CSCs and Sec62 in CRC, thereby stabilizing β-catenin signaling and promoting drug tolerance.^166,167^

Finally, signal amplification at the membrane and intracellular kinase nodes can increase pathway gain in CSCs. LGR6 promoted proliferation and metastasis through WNT/β-catenin signaling in triple-negative breast cancer (TNBC), consistent with a role in strengthening canonical output.^176^ In breast cancer, SIK2 phosphorylated LRP6 to activate WNT/β-catenin signaling and maintain stemness.^177^ Collectively, genetic lesions, non-canonical receptor wiring, and epigenetic/epitranscriptomic control create a highly adaptable WNT network that supports CSC persistence and therapy resistance across tumor contexts.

#### Notch signaling pathway

The Notch pathway mediates contact-dependent communication between adjacent cells and contributes to cancer progression, treatment response, lineage decisions, and the maintenance of stem-like tumor cell populations.^165,178,179^ In CSCs, Notch activation can promote self-renewal and sustained proliferation, but its net effect is highly context-dependent, reflecting ligand abundance, receptor usage, receptor trafficking, and crosstalk with pathways such as WNT, Hippo/YAP, and inflammatory signaling.^165,178,179^

Mammalian Notch signaling is initiated by four single-pass transmembrane receptors (Notch1-4) and five canonical membrane-bound ligands (JAG1, JAG2, DLL1, DLL3, DLL4) on a neighboring signal-sending cell.^179^ Notch receptors are synthesized as precursors and are processed during maturation (S1 cleavage) to form a heterodimeric receptor in which the negative regulatory region (NRR) keeps the receptor in an autoinhibited state at the cell surface. Ligand binding, typically in trans, between neighboring cells, induces a conformational change that exposes the S2 cleavage site, enabling ectodomain shedding by ADAM family metalloproteases, predominantly ADAM10, with ADAM17 contributing in some contexts.^179,180^ The remaining membrane-tethered fragment is then cleaved within the transmembrane region (S3 cleavage) by the γ-secretase complex. These sequential cleavages release the Notch intracellular domain (NICD) (Fig. 2b). NICD translocates to the nucleus and binds the DNA-associated transcription factor RBPJ/CSL, displacing co-repressors and recruiting co-activators such as MAML to assemble a transcriptional activation complex that induces canonical Notch target genes. This NICD/RBPJ/MAML module is the key output node that converts short-range receptor engagement into a sustained transcriptional program relevant to CSC maintenance and plasticity.^165,178,179^

Dysregulated Notch activity contributes to CSC persistence and phenotypic plasticity in several malignancies. In T-ALL, gain-of-function Notch1 alterations, including mutations that favor ligand-independent activation or prolong NICD stability, promote leukemia-initiating cell activity and leukemia maintenance.^181,182^ In colorectal cancer, CSC-enriched fractions show increased Notch pathway activity relative to non-CSC tumor cells, including elevated HES1 and JAG1 expression, and stromal/tumor crosstalk can further amplify Notch signaling in the tumor niche.^183^ In breast cancer, Notch signaling has been linked to ALDH-associated stem-like states and therapy tolerance. For example, Notch activation expands ALDH^high^ subpopulations and can be induced through inflammatory COX-2-EP4-PI3K/AKT wiring that feeds into Notch/WNT programs.^184,185^

Notch pathway amplitude is also set by receptor trafficking and degradation. The endocytic adaptor NUMB functions as a negative regulator by promoting Notch receptor internalization and degradation, thereby limiting NICD production.^186,187^ In gastric cancer, CD51 supports stemness by binding NUMB and blocking NUMB-mediated Notch1 degradation, enhancing Notch signaling and CSC persistence.^186^ Similarly, RAB4A has been described as an upstream regulator of cancer stemness that acts through the NUMB-Notch axis, illustrating how membrane trafficking can set the Notch-gain independently of ligand availability.^187^ These examples help explain why Notch output may remain high in CSCs even without large changes in ligand expression.

Notch transcriptional output can also coordinate with EMT-related programs. Notch activity has been associated with reduced epithelial features and enhanced invasive behavior through interactions with EMT-associated regulators and pathway crosstalk.^179^ The EMT transcription factor ZEB1 connects EMT and stemness to Notch by repressing stemness-inhibiting microRNAs, particularly the miR-200 family, thereby indirectly facilitating Notch activation and CSC survival in pancreatic and breast cancer models.^32,188^ Finally, CSC-enriched genes such as HIST2H2BF and STRAP have been reported to promote Notch activation by increasing NICD release, forming reinforcing loops that stabilize stem-like states.^165,189,190^

#### Hedgehog signaling pathway

The Hedgehog (Hh) pathway participates in tissue homeostasis, embryogenesis, regeneration, and contributes to tumor progression in several malignancies. The Hh family includes three homologous ligands, Sonic hedgehog (Shh), Indian hedgehog (Ihh), and Desert hedgehog (Dhh), which have distinct spatial and temporal expression patterns. Core pathway components consist of secreted Hh ligands, the Patched receptor PTCH, the transmembrane protein SMO, intermediate signaling molecules, and the downstream GLI transcription factors (Fig. 2c). Binding of Hh ligands to PTCH relieves inhibition of SMO, which promotes nuclear accumulation of GLI proteins and transcription of Hh target genes.^191^ GLI1, GLI2, and GLI3 mediate Hh responses as transcriptional activators or repressors and regulate processes such as cell growth, proliferation, and differentiation.

Hedgehog signaling, beyond its roles in embryonic development, has been linked to regulation of CSC self-renewal, EMT programs, and chemotherapy tolerance in several cancers.^192–194^ Dysregulated pathway activity influences CSC populations in leukemia, colorectal, lung, myeloma, and breast tumors.^195–200^ In CSCs, core components such as PTCH1, GLI1, GLI2, and SMO often show increased expression, and pathway output decreases upon differentiation.^196–199^ This enhanced activity is shaped by multiple regulators. In breast cancer stem cells characterized by CD44^+^CD24^-/low^Lin^-^, expression of GLI1, GLI2, and Bmi-1 are elevated compared to non-stem tumor cells, establishing the Hh–Bmi-1 pathway as a key regulator of their self-renewal and tumor-initiating capacity.^197^ Further supporting this, RUNX3-mediated degradation of GLI1 reduces CSC populations in colorectal cancer,^201^ while in medulloblastoma CSCs, the phosphatase WIP1 enhances GLI nuclear localization and protein stability, which are needed to sustain their undifferentiated state.^202^

Indeed, Hh signaling contributes to maintenance of molecular and functional stem-related traits in several cancers. Part of this effect involves transcriptional control of genes linked to pluripotency and self-renewal.^200^ An additional layer of regulatory complexity involves long non-coding RNAs (lncRNAs) that are functionally associated with the Hh signaling cascade. For example, Shh-GLI1 pathway-associated lncRNA-Hh has been shown to alter the expression of key pluripotency factors like OCT4 and SOX2, which support CSC survival and the maintenance of their undifferentiated state.^203^ This context-dependent reliance on Hh signaling is often exploited by cancers through oncogenic mutations. For example, in basal cell carcinoma and medulloblastoma, mutations in components of the Hh pathway, such as PTCH1 or SMO, lead to its constitutive activation and have been linked to the maintenance of a CSC phenotype.^204,205^ Taken together, current data indicate that Hh signaling functions as a context-dependent regulator of CSC maintenance.

#### Hippo signaling pathway

The Hippo pathway regulates organ size, tissue balance, and stem cell behavior, and its alteration contributes to tumor development. It also shapes the biology of CSCs, which sustain tumor initiation, metastatic spread, and treatment tolerance. The canonical route is formed by a kinase sequence that includes MST1/2 together with LATS1/2. When this sequence is active, MST1 and MST2 phosphorylate LATS1 and LATS2, which then phosphorylate the transcriptional co-activators YAP and TAZ (Fig. 2d). Phosphorylated YAP and TAZ remain in the cytoplasm and are subsequently degraded. Several parallel kinases, including members of the MAP4K family, also activate LATS proteins and help maintain YAP and TAZ in an inactive state.^206^ When Hippo signaling is reduced, unphosphorylated YAP and TAZ enter the nucleus and cooperate with TEAD transcription factors to control genes involved in cell division, survival, and stem-like behavior.

Evidence from breast cancer first established that TAZ activity supports renewal and tumor-forming capacity in CSCs.^207^ TAZ protein levels are elevated in CSC enriched populations and in poorly differentiated tumors, and forced expression of TAZ can confer renewal capacity on non-CSCs. Loss of polarity components, such as Scribble, disrupts the restraint on TAZ imposed by the core kinases. This loss of polarity, which often accompanies EMT, therefore promotes TAZ activation.

Subsequent work in multiple tumor types has clarified that YAP and TAZ create a transcriptional program that stabilizes CSC traits. For example, YAP inactivation was shown to abolish adenoma formation in a mouse model for colon cancer where the YAP-driven regenerative pathway is essential for the progression of early tumor-initiating cells.^208^ YAP and TAZ also contribute to treatment tolerance and metastatic behavior. Elevated TAZ can reduce sensitivity to paclitaxel in breast cancer, in association with CYR61 and CTGF induction through TEAD binding.^209^ YAP and TAZ activity influences resistance to targeted therapies as well, in part by increasing anti-apoptotic factors such as BCL-XL.^210^ Overall, Hippo signaling, through YAP and TAZ, shapes renewal capacity, metastatic behavior, and treatment tolerance within CSC pools. Regulation of these two co-activators involves multiple inputs from polarity networks, kinase modules, and transcriptional feedback circuits. Current evidence indicates that inhibitors directed at the YAP and TAZ interaction with TEAD may offer an approach for limiting CSC-driven tumor persistence and for improving responses to existing treatments.

### Microenvironment and stress-regulated programs

**Fig. 3:**
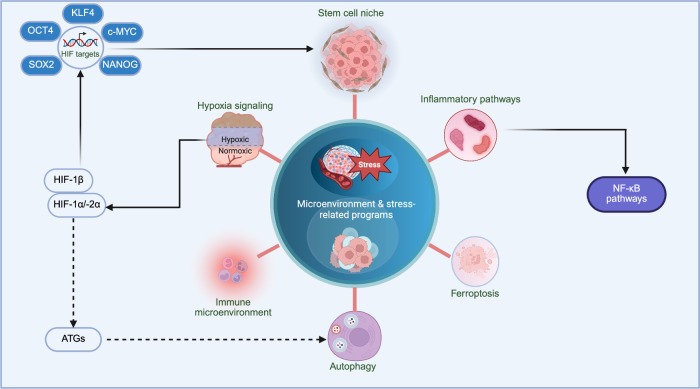
Microenvironmental and stress-regulated pathways that support CSC behavior. Hypoxia stabilizes HIF-α subunits, which dimerize with constitutively expressed HIF-1β/ARNT and activate hypoxia-responsive gene programs that influence stem-cell niches. Inflammatory cues activate NF-κB routes that support self-renewal, EMT-linked traits and immune modulation. Ferroptosis-defense programs protect CSC-like cells from lipid peroxidation through system xc-/xCT, glutathione-linked antioxidant pathways and related lipid-repair mechanisms. Autophagy uses ATG proteins, lysosome biogenesis, mitophagy and selective cargo turnover to maintain stress-adapted stem-like states. Immune and stromal components of the niche integrate signals from these pathways to support CSC maintenance across tumor types. Created in BioRender. Uddin, K. (2026) https://BioRender.com/jbnalll

CSC behavior is shaped not only by developmental routes but also by signals that arise from local stress, metabolic strain, immune activity, and niche composition. These conditions activate pathways that support survival during hypoxia, inflammation, nutrient shortage, and therapeutic pressure (Fig. 3).

#### NF-κB signaling pathway

NF-κB is a family of transcription factors that links inflammatory signals and cellular stress to gene programs controlling survival, proliferation, and cell state plasticity. In many cancers, NF-κB activity is enriched in CSC-like fractions and can support tumor initiation, therapy tolerance, and microenvironmental remodeling.^211^ Mechanistically, NF-κB signaling is commonly discussed as two interconnected modules, canonical and non-canonical, that differ in upstream triggers, core kinases, and the NF-κB dimers they activate.^211^

Canonical NF-κB is typically activated by cytokines and innate immune cues, such as TNF-family signaling and Toll-like receptors (TLRs) inputs. These stimuli converge on the IKK complex, where IKKβ is the dominant catalytic subunit and NEMO (IKKγ) serves as an essential scaffold. Activated IKKβ phosphorylates IκBα, the inhibitory protein that retains NF-κB dimers in the cytoplasm. IκBα phosphorylation targets it for proteasomal degradation, allowing the RelA/p65-p50 dimer to translocate to the nucleus and induce transcription.^211^ Non-canonical NF-κB is engaged by a more restricted set of receptors and depends on stabilization of NF-κB-inducing kinase (NIK), which activates IKKα and drives proteolytic processing of p100 (NF-κB2) into p52, enabling nuclear accumulation of RelB-p52 complexes. Although presented as separate branches, canonical and non-canonical components often cooperate in CSC biology, and pathway output depends on which NF-κB subunits are available, which upstream signals dominate, and how NF-κB interacts with developmental and epigenetic circuits.^211^

Studies in epithelial tissues illustrate how NF-κB can be permissive for stem-like behavior and, under sustained activation, become instructive for tumor initiation. In the intestine, chronic NF-κB activity increases WNT pathway output and enables differentiated epithelial cells to dedifferentiate and acquire tumor-initiating capacity, whereas genetic loss of RelA/p65 restricts expansion of crypt stem-like cells.^47^ A closely related principle has been shown in lung adenocarcinoma (LUAD), where sustained NF-κB activation allows mutant alveolar type II epithelial cells to resist differentiation and instead co-opt a regeneration-like program that supports tumor initiation.^212^ Inhibiting NF-κB promotes restoration of a more normal lineage state. At later LUAD stages, additional regulators can amplify this axis. For example, GJB2 enhances nuclear accumulation of p65, induces SOX2, and expands CSC-associated fractions marked by CD133 and CD44, consistent with NF-κB acting upstream of a stemness transcriptional program during progression.^213^ Together, these data position canonical NF-κB not only as a stress-response pathway but also as a determinant of whether injured or mutant epithelial cells commit to differentiation versus adopt stem-like, tumor-propagating states.

In glioblastoma, CSC maintenance has been strongly linked to RelB-containing NF-κB activity and its integration with niche remodeling. Interferon-induced protein 35 (IFI35) was shown to regulate NF-κB signaling in glioblastoma stem cells by promoting p105-to-p50 processing and cooperating with RelB, thereby supporting self-renewal while also recruiting tumor-associated macrophages, an example where NF-κB output couples intrinsic stemness with extrinsic microenvironment shaping.^214^ Metabolic stress can further feed into this circuitry. Lactate-driven histone lactylation was reported to induce the lncRNA LINC01127, which activates a MAP4K4/JNK/NF-κB route that sustains glioblastoma stem-like behavior, placing NF-κB downstream of tumor metabolism and epigenetically influenced transcriptional reprogramming.^215^ In this setting, NF-κB is best viewed as an integrator that converts metabolic and microenvironmental pressures into durable transcriptional states compatible with self-renewal and niche adaptation.

Breast cancer provides additional examples of NF-κB cooperation with chromatin-linked regulators and immune-evasion programs. In CSC-enriched breast cancer cells, the lncRNA HOTAIR associates with p65, and HOTAIR suppression reduces mammosphere formation and stemness-associated transcription, supporting a model in which canonical NF-κB collaborates with lncRNA-dependent regulatory complexes to maintain stem-like states.^216^ In parallel, a BRD4-nuclear PD-L1-RelB circuit was reported to sustain CSC-associated phenotypes, including ALDH^high^ and CD44^+^/CD24^−^ features, highlighting that non-canonical NF-κB subunits can link stemness maintenance with immune checkpoint biology through non-membrane roles of PD-L1.^217^ These findings emphasize that in some tumors NF-κB output is reinforced not only by inflammatory ligands but also by epigenetic readers and non-classical nuclear signaling nodes.

Comparable NF-κB-centered logic appears across additional malignancies. For example, in colorectal cancer, interventions that reduce TLR4/NF-κB signaling suppress sphere formation and diminish EMT-associated behavior, consistent with NF-κB supporting survival and plasticity programs that enrich CSC-like states.^218^ In ovarian cancer, the glycolytic regulator PFKFB3 increases NF-κB pathway output and maintains ALDH^+^ and CD44^+^ populations.^219^ PFKFB3 inhibition reduces metastasis and drug tolerance, linking metabolic control to NF-κB-dependent stemness. In hepatocellular carcinoma, inflammatory TNFα activates NF-κB, reduces miR-497, and increases the stemness factor SALL4, promoting sphere formation and migration, an explicit mechanistic bridge from chronic inflammation to a CSC gene program.^220^ In AML, limiting persistent NF-κB signaling can enhance bortezomib sensitivity in CD34^+^ AML cells, supporting NF-κB as a therapeutic vulnerability in leukemia stem-like compartments.^221^ Overall, NF-κB functions as a signal-integration hub that translates cytokine/TLR inputs, metabolic stress, and pathway crosstalk into transcriptional programs that can reinforce CSC traits, promote EMT-like plasticity, and coordinate immune- and niche-related adaptations.

#### Hypoxic cell signaling

Hypoxic cell responses regulated by HIF-1α and HIF-2α can reshape transcription, metabolism, and microenvironmental interactions in ways that support CSC survival.^48^ The functional roles of HIFs are to some extent overlapping, since the combined loss of genes encoding HIF-1α and HIF-2α suppresses stem-related programs more strongly than loss of either alone.^222^ The stabilization of the HIF-α proteins is dependent on tissue context, oxygen tension, and developmental stage.^223,224^ In cancer, the hypoxia response machinery can be conserved or constitutively expressed.^225^

A major output of hypoxia-response machinery is the reinforcement of developmental pathways. In glioma stem-like cells, a HIF-1α and STAT3 route induces Vasorin, which limits Numb-driven Notch1 degradation and maintains Notch activity.^226^ A similar pattern appears in prostate cancer, where induction of the hypoxic response increases Notch1 expression, sphere formation, and invasion.^227^ In contrast, suppression of HIF-1α reverses these changes.^227^ Hypoxia also modulates WNT/ β-catenin-related behavior. In colorectal cancer, glycolytic activity under physiologically low oxygen conditions increases β-catenin lactylation, stabilizing β-catenin and supporting CSC traits.^228^ Similarly, in breast cancer, the lncRNA RBM5AS1 is upregulated under hypoxia and sustains WNT/β-catenin signaling.^229^ However, these responses appear to be lineage-dependent, as hypoxia increases β-catenin levels and ALDH-high fractions in squamous NSCLC while reduces both in adenocarcinoma.^230^

Adaptations driven by the hypoxic response are further supported by autophagy-related mechanisms. In glioblastoma, hypoxia-induced Galectin-8 interacts with the Ragulator and Rag complex, suppresses mTORC1 activity, and increases TFEB-driven lysosome biogenesis, thereby maintaining stem-related traits.^231^ HMGB1 induction also contributes to survival, and its loss reduces expression of stemness markers and limits self-renewal through reduced RAGE signaling.^232^ Additional support is provided by chromatin and metabolic reprogramming. HIF-1α cooperates with FACT and the RNF20/RNF40 ligase complex to establish hypoxia-responsive chromatin states.^233^ Hypoxia also induces CTCF through TET2-mediated demethylation and direct HIF-1α binding, promoting EMT-associated plasticity.^234^ In glioblastoma, reduced DHFR and increased MAT2A enable a shift in one-carbon metabolism that supports folate-independent growth.^235^ Moreover, hypoxia decreases sensitivity to cuproptosis, a copper-dependent form of cell death, whereas loss of HIF-1α restores susceptibility to this pathway.^236^ Altered oxygen dynamics within the local niche not only affect tumor cell fitness and adaptive responses but also reshape the macrophage landscape. For example, in M2-like macrophages, TREM1 increases TGF-β2 production and activates TGFβR-SMAD2/3 signaling in glioblastoma stem-like cells, promoting a mesenchymal phenotype.^237^ Furthermore, long-term hypoxic organoid co-culture models show similar microenvironmental remodeling that favors invasion.^238^

These interconnected processes suggest potential combinatorial therapeutic routes to disrupt HIF-dependent CSC maintenance and adaptive survival programs. One avenue is to combine therapy with direct inhibition of HIF-2α. Others include strategies that attenuate HIF-1α-driven stress programs, for example those that confer resistance to cuproptosis, and interventions aimed at downstream circuits such as hypoxia-Notch or hypoxia-WNT coupling. Tumor lineage and microenvironmental context should guide such efforts, since hypoxic responses can differ across cancer types.

#### Ferroptosis defense signaling

Ferroptosis is a regulated form of cell death driven by iron-dependent lipid peroxidation, in which polyunsaturated fatty acid (PUFA)-containing membrane phospholipids accumulate damaging lipid hydroperoxides.^239^ CSC-like tumor cells frequently avoid ferroptosis by strengthening antioxidant systems that limit the formation of lipid peroxides and repair them once formed.^240^ Across many tumor contexts, these defenses form a layered network rather than a single pathway, which helps explain why CSC-like populations can persist under oxidative and therapeutic stress. A major ferroptosis defense module begins at the plasma membrane with system xc^-^, a cystine/glutamate antiporter composed of the catalytic subunit SLC7A11 (xCT) and its partner SLC3A2. Imported cystine is reduced intracellularly to cysteine, which is required for synthesis of glutathione (GSH). GSH in turn fuels glutathione peroxidase 4 (GPX4), a lipid repair enzyme that reduces phospholipid hydroperoxides to non-toxic lipid alcohols, thereby blocking the lethal buildup of lipid peroxides and preventing ferroptosis.^241^

CSC-like states can enhance this axis through surface scaffolds and stress-responsive regulation. A key example is the CD44 variant (CD44v), which associates with and stabilizes xCT at the membrane, increasing cystine uptake, raising intracellular GSH, and improving redox control in stem-like cancer cells.^98^ This mechanism also has translational relevance. The xCT inhibitor sulfasalazine has been clinically evaluated in combination with cisplatin in CD44v-positive gastric cancer, illustrating the feasibility of targeting this membrane entry point in CD44v-enriched tumors.^242^ Functionally, additional tumor-specific regulators can tune the same core defense. In esophageal CSC-like cells, Hsp27 promotes an xCT/GPX4-high state through suppression of p53, reducing lipid peroxidation and preserving stemness.^243^ In colorectal cancer, CSC-enriched populations show elevated SLC7A11 and GSH, and genetic or pharmacologic suppression of SLC7A11 induces ferroptosis and inhibits CSC-associated progression.^244^ Together, these studies position the CD44v/xCT/GSH/GPX4 module as a recurrent, targetable ferroptosis defense pathway in CSC-like compartments.

Ferroptosis sensitivity is strongly influenced by membrane composition, because PUFA-containing phospholipids are preferred substrates for lipid peroxidation. CSC-like cells can reduce this liability by shifting membranes toward monounsaturated fatty acids (MUFAs), which are less prone to peroxidation. Stearoyl-CoA desaturase 1 (SCD1) generates MUFAs that can displace PUFAs within phospholipids and thereby lower the pool of oxidizable lipids. In ovarian cancer, including CSC-associated settings, SCD1 activity protects cells from ferroptosis, whereas SCD1 inhibition triggers lethal lipid damage.^245^ Mechanistic work further showed that exogenous MUFAs or MUFA incorporation programs promoted by ACSL3 can drive a ferroptosis-resistant membrane state by replacing peroxidation-prone PUFAs in phospholipids.^246^ These lipid-defense programs can be reinforced by oncogenic context. In STK11/KEAP1 co-mutant lung adenocarcinoma, ferroptosis-protective lipid remodeling is enhanced, creating dependence on lipid-handling enzymes including SCD1 and additional detoxification modules such as AKR1C enzymes.^247^ This illustrates how genetic background can lock in ferroptosis-resistant lipid states that are particularly compatible with therapy-tolerant or stem-like phenotypes.

CSC-like cells can also rely on antioxidant systems that suppress lipid peroxidation independently of GPX4, providing redundancy when one defense arm is inhibited. One key pathway is FSP1 (AIFM2), which regenerates reduced coenzyme Q (CoQ/ubiquinol) at membranes. Reduced CoQ functions as a radical-trapping antioxidant that intercepts lipid peroxyl radicals and suppresses ferroptosis without requiring GSH/GPX4.^248^ A second, compartment-specific defense operates in mitochondria via DHODH, which reduces ubiquinone to ubiquinol and protects mitochondrial membranes from lipid peroxidation.^249^ Importantly, DHODH inhibition can kill GPX4-low tumors and can also enhance the impact of system xc^-^ blockade when GPX4 is present, consistent with these systems acting as partially compensatory layers. A third GPX4-independent route involves GCH1 and its product tetrahydrobiopterin (BH4), which creates an antioxidant pool and promotes lipid remodeling that limits lipid peroxidation.^250^ Collectively, FSP1/CoQ, DHODH/CoQ, and GCH1/BH4 provide alternative ways to restrain lipid peroxide accumulation and can help CSC-like states survive under high oxidative stress.

Ferroptosis defenses are closely linked to drug-tolerant persistence, a state that often retains tumor-initiating capacity. Drug-tolerant persister cells show a pronounced dependence on GPX4, and GPX4 inhibition can eliminate persisters and reduce relapse in vivo.^241,251^ Consistent with this, glioblastoma studies indicate that specific developmental/malignant states can display a GPX4-dependent ferroptosis vulnerability, suggesting that ferroptosis sensitivity may map to discrete stem-like or regenerative states rather than to all malignant cells uniformly.^252^ Ferroptosis can also be triggered by manipulating iron handling. Activation of lysosomal iron can induce ferroptosis in therapy-resistant cancer cells with high CD44 and increased iron load, features frequently associated with CSC-like states.^253^ In summary, ferroptosis resistance in CSC-like populations is typically achieved through redundant and layered defenses. This redundancy suggests rational combination strategies, such as targeting system xc^-^ or GPX4 while simultaneously inhibiting compensatory modules such as DHODH or SCD1, to more effectively disrupt CSC persistence and therapy-tolerant reservoirs.

#### Autophagy-related signaling

CSCs across solid and hematologic tumors exploit the autophagy-lysosome system to withstand stress, adapt metabolism, and persist during therapy. In glioblastoma stem-like cells, hypoxic conditions induce a Galectin-8/mTORC1/TFEB signaling axis promoting lysosomal biogenesis and autophagy to sustain self-renewal capacity.^231^ Furthermore, the chaperone-dependent E3 ubiquitin ligase STIP1 homology and U-box-containing protein 1 (STUB1) binds serine-phosphorylated TFEB, directing it for proteasomal degradation and thereby regulating TFEB-driven autophagy.^254^ Chaperone-mediated autophagy also contributes to glioblastoma stem cell maintenance. This process is elevated in glioblastoma stem-like cells through the receptor LAMP2A and is required for tumor formation. Suppression of chaperone-mediated autophagy reduces stem-related transcription and limits tumor growth.^255^ In addition, pharmacologic lysosome inhibition with lucanthone depletes the glioblastoma stem cell pool and enhances the effect of temozolomide in vivo.^256^

Autophagy interacts with metabolic shifts in glioblastoma as well. Patient-derived models show a reversible interplay between autophagy and glycolysis that enables transitions between senescent and stem-like states during stress, linking metabolic adaptation to the maintenance of renewal capacity.^257^ In some settings, autophagy can instead restrict stem-like traits. Glutamine withdrawal promotes degradation of SIRT3 through autophagy, which reduces CD133 expression and limits renewal.^258^ These observations indicate that the influence of autophagy depends on the upstream trigger and the specific cargo targeted for degradation.

Outside the brain, epithelial CSCs also use autophagy to regulate their behavior. In head and neck squamous cell carcinoma, autophagy supports stem-related features through a FOXO3 and SOX2 pathway, and autophagy inhibition reduces sphere growth and tumor initiation.^259^ Ovarian CSCs show high levels of mitophagy via the PINK1/Parkin pathway, which supports their resistance to PARP inhibitors; astragalus polysaccharides inhibit mitophagy through this signaling axis, thereby increasing sensitivity to PARP inhibition, reducing self-renewal capacity, and decreasing cell viability.^260^ Autophagy also removes ID1 in this setting, shaping transcriptional profiles linked to therapy tolerance.^261^ Leukemic stem cells also depend on autophagy to manage low oxygen and mitochondrial stress. In primary AML, autophagy is elevated in CD34-positive and CD38-negative cells. Blocking autophagy reduces their survival in hypoxia and decreases leukemia burden in xenografts, supporting combined use of chemotherapy with autophagy inhibitors.^4,262^

Overall, these studies place macro-autophagy, TFEB- and TFE3-driven lysosome formation, chaperone-mediated autophagy through LAMP2A and HSC70, and selective mitophagy through PINK1 and Parkin at the center of CSC persistence. Therapeutic options include suppressing adaptive autophagy or chaperone-mediated autophagy to limit drug tolerance or directing autophagy toward degradation of proteins that support renewal. Matching the intervention to the trigger, cargo, and tumor lineage remains a key consideration for effective CSC targeting.

### Growth factor and cytokine-driven cascades

CSC behavior is strongly shaped by extracellular cues that signal through growth factor receptors and cytokine-linked pathways. These inputs regulate renewal capacity, adaptive survival, and lineage plasticity by activating intracellular routes that include SMADs, PI3K or AKT, MAPK, PKC, and JAK or STAT. The following sections outline how these cascades integrate microenvironmental signals with transcriptional and metabolic control to support CSC maintenance across diverse tumor settings.

#### Transforming growth factor beta (TGF-β) signaling pathway

The transforming growth factor beta (TGF-β) signaling pathway is a complex signaling network that governs multiple cellular processes and plays a central role in maintaining tissue homeostasis. In cancer, however, TGF-β signaling possesses a dual role: acting as a tumor-suppressor in early stages while promoting tumor progression, metastatic dissemination, and chemoresistance during later stages of malignancy.^263,264^

TGF-β represents a family of soluble proteins including three TGF-β receptor ligands: TGF-β1, TGF-β2, TGF-β3, bone morphogenetic proteins (BMPs), Activins (Acts), growth differentiation factors (GDFs), Nodals, and the müllerian inhibiting substance (MIS). These ligands signal through a heteromeric complex of type I and II transmembrane serine/threonine kinase receptors. Mechanistically, in canonical signaling, ligand binding to the type II receptor facilitates recruitment and phosphorylation of the type I receptor, which in turn phosphorylates receptor-regulated SMADs (R-SMADs: SMAD2 and SMAD3 for TGF-β; SMAD1, SMAD5, and SMAD8 for BMPs). Activated SMADs form a complex with the common mediator SMAD4 and translocate into the nucleus where they modulate the transcription of target genes.^264^ In contrast, the non-canonical pathway is independent of SMAD proteins and involves activation of signaling cascades such as Rho-like GTPases, p38 MAPK, jun N-terminal kinase (JNK), mitogen-activated protein kinase (ERK or MKK), and phosphoinositide 3-kinase (PI3K)-AKT axis.

Within CSC biology, TGF-β functions as a key regulator of self-renewal, plasticity, and migratory behavior. Several studies in breast cancer show that TGF-β promotes stem-associated subpopulations such as CD49F^high^ and CD61^high^ cells in breast CSCs, as well as the CD44^+^ subset in both breast and gastric CSCs.^265–267^ For example, the helix-loop-helix transcriptional regulator ID1 induces EMT-independent tumor-initiating properties in human mammary cells.^268^ EMT-related transcription factors induced by TGF-β, such as SNAIL, SLUG, TWIST, and ZEB1 or ZEB2, help generate stem-like states, as demonstrated in EMT-driven CSC models where TGF-β/SMAD activity preserves invasive plasticity.^9,269^ Colorectal CSC models further support this pattern. Microenvironmental TGF-β promotes the dormancy of LGR5-positive colonic stem cells,^270^ and induces the secretion of factors such as IL-11 that enhance survival and colonization of metastatic cells.^271^

In pancreatic ductal adenocarcinoma, CD44⁺CD24⁺ALDH⁺ cells behave as CSCs with high tumor-initiating capacity.^272^ Furthermore, in PANC‑1 cells, activation of the TGF‑β/Smad3 pathway increases sphere formation, migration and the proportion of CD24⁺, CD44⁺ and CD133⁺ cells, linking TGF‑β directly to CSC traits, proliferation and migration.^273^ Glioblastoma models also showed a related pattern. Autocrine TGF‑β/SMAD signaling in glioma‑initiating cells induces SOX2/SOX4 and maintains sphere-forming capacity and tumorigenicity.^274^ Beyond direct effects on tumor cells, TGF‑β also shapes the surrounding microenvironment. For example, TGF‑β2 upregulates MMP‑2 and reduces TIMP‑2 expression, which facilitates extracellular matrix degradation and local invasion.^275–277^ Collectively, current work supports a model in which TGF-β couples CSC maintenance to microenvironmental remodeling in several tumor contexts.

#### Receptor tyrosine kinase (RTK)-driven pathways

**Fig. 4:**
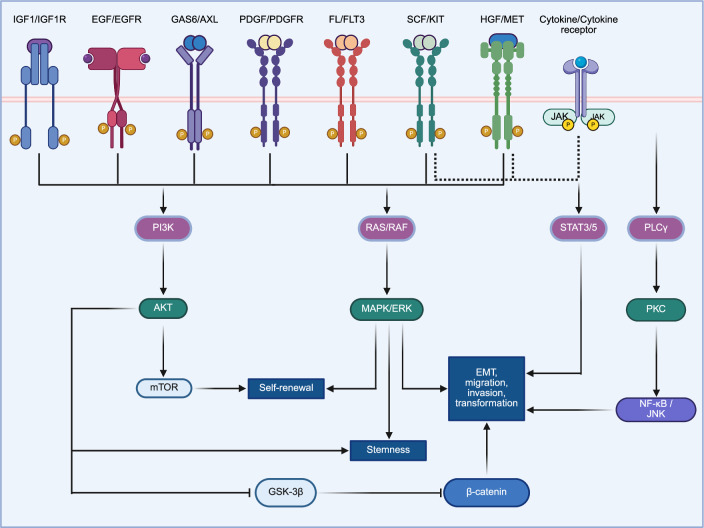
Growth factor- and cytokine-driven RTK networks that regulate CSC signaling outputs. RTKs and cytokine-linked receptors are activated by their ligands, including EGFR, AXL, PDGFR, IGF-1R, MET, FLT3 and KIT. These receptors converge on intracellular modules such as PI3K, AKT, mTOR, MAPK/ERK, STAT3/STAT5, PLCγ, PKC, NF-κB and JNK. Coordinated activity within these modules regulates renewal capacity, β-catenin stabilization, GSK3β control, EMT-associated behavior, migration, invasion and transformation. Where line styles are used, they indicate direct receptor-proximal signaling versus indirect pathway crosstalk or downstream phenotypic outputs. Created in BioRender. Uddin, K. (2026) https://BioRender.com/mddodwh

RTK networks shape CSC identity, plasticity, and treatment resilience in several tumor types. Several RTKs including EGFR, PDGFR, IGF-1R, AXL, MET, FLT3 and KIT play important roles in CSC biology (Fig. 4). For example, the EGFR splice variant (EGFRx) is selectively expressed by glioblastoma stem cells (GSCs) and maintains their self‑renewal and tumor‑initiating capacity, suggesting that RTK isoform architecture can encode stemness programs.^278^ Mechanistically, EGFR couples to canonical transcriptional nodes (STAT3/STAT5) to stabilize stem cell states; a positive‑feedback loop between PARN and EGFR-STAT3 supports GSC proliferation and serial tumor propagation, and its interruption curtails GSC self‑renewal.^279^ Earlier studies showed that the common truncation mutant EGFRvIII drives CSC traits through STAT3 signaling.^280^ Ligand context can further modulate EGFR outputs as in EGFR‑amplified GBM. Constitutive activation of EGFR promotes invasion via a TAB1-TAK1-NF‑κB-EMP1 cassette, whereas ligand‑activated EGFR switches to a more proliferative, less invasive state and acts as a context‑dependent tumor suppressor.^281^

Beyond transcription, RTK signaling integrates epitranscriptomic control to lock in CSC fitness. Activated EGFR suppresses m^6^A methylation in GSCs by promoting ALKBH5 nuclear retention via SRC‑dependent phosphorylation.^282^ Genetic or pharmacologic EGFR blockade restores m^6^A and impairs stemness, highlighting a tractable EGFR-m^6^A-ALKBH5 axis.^282^ PDGFR signaling also intersects with m^6^A machinery. In CSCs, PDGF-PDGFR signaling increases global m^6^A by inducing early growth response 1 (EGR1) transcription, which in turn upregulates the writer methyltransferase-like 3 (METTL3).^283^ METTL3 adds m^6^A to optineurin (OPTN), thereby restraining mitophagy and supporting GSC self‑renewal. Together, these data place RTK-m^6^A coupling as an added control layer for CSC maintenance in glioblastoma.

TAM‑family RTKs, in particular AXL, act as recurrent CSC‑supportive receptors. In patient‑derived GSC models, AXL activation promotes survival, proliferation, and expression of mesenchymal and immunomodulatory programs, whereas genetic knockdown or pharmacologic inhibition with the small‑molecule inhibitor BGB324 reduces GSC growth, alters cytokine output, and extends survival of intracranial xenografts.^284^ Outside the brain, AXL marks a multipotent population in the normal mammary epithelium and is enriched in basal‑like and mesenchymal‑type breast cancers. Functional assays demonstrate that AXL‑positive mammary cells have increased colony formation, multilineage differentiation capacity, and tumor initiation, while AXL inhibition reduces these stem‑like behaviors.^285^ Complementing these mechanistic data, unbiased kinome‑wide screening nominated AXL as a driver of mesenchymal/therapy‑resistant states with CSC features in GBM, reinforcing AXL biology as an actionable convergent node.^286^

The MET/HGF pathway also marks and controls GSC subsets. Analyses of patient glioblastoma specimens and matched GSC lines show that MET‑positive GSCs form larger neurospheres, initiate tumors more efficiently in vivo, and are more sensitive to MET inhibition than MET‑negative counterparts, indicating a MET‑dependent GSC subpopulation.^287,288^ Ephrin receptors participate in this RTK hierarchy as well. For example, EphA2 is overexpressed in human glioblastoma tumor‑propagating cells, and EphA2^high^ cells show increased self‑renewal and tumorigenicity. Genetic or ligand‑induced EphA2 downregulation drives differentiation and reduces intracranial tumor formation, placing EphA receptors alongside EGFR, AXL, and MET as GSC regulators.^289^

RTK activity also feeds into metabolic programs that favor GSC maintenance. Single‑cell RNA sequencing and metabolomics of EGFR‑activated glioblastoma identified an RTK/fatty‑acid/mevalonate gene signature; combined inhibition of EGFR/AKT and the mevalonate pathway reprograms energy metabolism, reduces neurosphere growth, and synergizes with temozolomide in vivo.^290^ These data link EGFR‑driven signaling to lipid and cholesterol biosynthesis as a metabolic support axis for stem‑like cells.^290^

Spatial and niche-driven cues can shift RTK balance toward or away from CSC states. Spatial/single‑cell profiling of gastric cancer delineated CSC niches in which amphiregulin (AREG)-ERBB signaling (notably ERBB2/HER2) and RTK ligand gradients enforce stemness programs and therapeutic tolerance, suggesting that RTK ligands within the niche are as consequential as receptor genetics.^291^ In GBM, EGFR amplification/mutation cooperates with connexin‑43-derived peptides and EGFR inhibitors to reshape GSC kinetics and treatment response, illustrating how microenvironmental and pharmacologic contexts intersect with RTK status in stem‑like cells.^292^

Across multiple epithelial cancers, IGF‑1R links growth factor input to transcriptional and epigenetic circuits that sustain CSC pools. In TNBC, insulin/IGF signaling through IGF‑1R and IRS2 activates PI3K and stabilizes MYC, promoting mammosphere formation and CSC frequency.^293^ IRS2 loss or pharmacologic blockade of IGF‑1R/PI3K reduces CSC‑associated phenotypes, while MYC stabilization partially rescues these defects. A separate study using patient‑derived breast CSC lines showed that IGF‑1R activity maintains YAP expression and nuclear localization. IGF‑1R inhibition decreases YAP, ALDH activity, mammosphere formation, and tumor initiation, whereas IGF‑1 drives nuclear YAP; in turn, YAP increases IGF‑1 transcription, establishing a reinforcing IGF‑1-IGF‑1R-YAP loop.^294^ These studies place MYC and YAP as nodal transcription factors downstream of IGF‑1R in breast CSCs.

IGF‑1R also connects to EMT and stem‑like traits. In cutaneous squamous cell carcinoma, IGF‑1R signaling is required for microenvironment‑induced epithelial-mesenchymal plasticity (EMP) and mesenchymal state acquisition, in part through induction of integrin αV (ITGAV).^295^ ITGAV expression labels lesions with higher relapse risk, and IGF‑1R or ITGAV targeting reduces migration, invasion, and expression of mesenchymal markers. These data link IGF-1R to dynamic EMT/EMP programs that are closely intertwined with CSC states.

IGF-1R shapes a FOXC1-centered transcriptional loop in esophageal squamous cell carcinoma. IGF-1 increases FOXC1 through PI3K or AKT and MEK or ERK-linked NF-κB activity, and FOXC1 increases CBX7 and IGF-1R transcription, raising CD44 and CD133 and promoting renewal and drug tolerance.^296^ This IGF-1/IGF-1R-FOXC1/IGF-1R positive feedback loop illustrates how IGF-1R signaling can be embedded into CSC-specific transcriptional circuits that reinforce stemness and therapy resistance. Liver and biliary tumors rely on IGF-1R in a similar manner. In hepatocellular carcinoma, IGF-1R promotes sphere formation, increases CD90- and CD133-population, and supports growth and metastatic spread, while IGF-1R inhibition lowers CSC frequency in xenografts.^297^ Kinase‑inhibitor library screening on tumorsphere cultures of intrahepatic cholangiocarcinoma, identified the IGF‑1R/PI3K/AKT axis as a major dependency where IGF‑1R knockdown or inhibitors reduced AKT activation, sphere viability, and xenograft growth, indicating that IGF‑1R supports cholangiocarcinoma CSC maintenance.^298^ These studies argue that IGF-1R is not merely a generic growth receptor but a central node for sustaining liver and biliary CSC pools.

IGF‑1R can also contribute to adaptive radio-resistance. Fractionated irradiation of GSCs resulted in chronic IGF‑1R upregulation and slowed baseline proliferation through FoxO3a activation yet improves survival and self‑renewal under radiation stress, while IGF‑1R inhibition restores radiosensitivity and reduces clonogenic survival.^299^ Complementary work showed that ionizing radiation triggers extracellular vesicle export of miR‑603, which normally represses IGF‑1, IGF‑1R, and the DNA repair gene MGMT.^300^ Loss of intracellular miR‑603 de‑represses IGF‑1/IGF‑1R, maintains CSC markers, and increases resistance to radiation and alkylating agents, whereas restoring miR‑603 reduces IGF‑1/IGF‑1R expression and resensitizes cells to therapy.

Class III RTKs FLT3 and KIT extend this theme to hematopoietic CSC systems. FLT3 is highly expressed on hematopoietic stem and progenitor cells, and its ligand (FLT3L/FL) is produced by bone‑marrow stromal and immune cells; together with SCF and other cytokines, FLT3L drives expansion of early CD34⁺ compartments and dendritic‑cell precursors.^301,302^ Activating FLT3 mutations, most often FLT3‑ITD, occur in roughly one third of AML cases and promote constitutive STAT5, RAS/MAPK and PI3K/AKT signaling that supports leukemic stem‑cell survival.^303^ Functional xenograft work with CRISPR‑mediated FLT3 knockout has shown that FLT3 is required for maintenance and propagation of FLT3‑ITD⁺ leukemic stem cells, whereas normal human HSCs retain long‑term multilineage reconstitution after FLT3 deletion, placing FLT3 in a relatively selective CSC‑dependency category.^304^ Genetic and multi‑omics studies further indicated that chronic FLT3‑ITD activity imprints transcriptional and metabolic states that preserve leukemia stem cell (LSC) pool, and that autophagy and mitochondrial metabolism interact with FLT3 signaling to sustain relapse‑initiating cells.^305,306^

In parallel, the SCF/KIT circuit couples microenvironmental cues to CSC traits in multiple solid and hematologic tumors. SCF produced by perivascular and stromal niche cells binds KIT (CD117) on HSCs to support proliferation, survival and homing, and similar SCF/KIT interactions operate in tissue stem‑cell compartments in prostate and other epithelia.^307,308^ Oncogenic KIT activation, either via gain‑of‑function mutations or autocrine SCF production, is a key driver in gastrointestinal stromal tumor (GIST) and is also present in subsets of AML, melanoma and other cancers. Co‑expression of SCF and mutant KIT in GIST supports autocrine growth and contributes to reduced sensitivity to imatinib, while SCF‑targeted conjugates such as SCF‑DM1 selectively deplete KIT‑driven cells in xenograft models.^309,310^ Work across tumor types indicates that KIT can mark tumor‑initiating fractions and that SCF/KIT signaling supports self‑renewal, EMT/EMP programs, metastatic spread and resistance to TKIs, placing SCF/KIT alongside EGFR, AXL and MET as an RTK axis that stabilizes CSC features in selected contexts.^308,311^

Protein kinase C (PKC), a family of ten protein serine/threonine kinases,^312^ intersects with several RTK pathways. RTKs can activate PKC isoforms through phospholipase C/D (PLC/PLD), and reciprocal feedback regulation may also occur. Classical biochemical work mapped PKC‑dependent phosphorylation to serines 741 and 746 within the KIT kinase‑insert region.^313^ These residues account for most SCF‑stimulated KIT serine phosphorylation in intact cells and modulate subsequent signaling. In SCF/KIT cascades, PLCγ activation has been reported but may be context-dependent, while PLD-dependent lipid signaling may also contribute to PKC engagement.^311^ Together, these pathways can promote diacylglycerol-dependent PKC activation, linking KIT signaling to feedback phosphorylation events that modulate survival, proliferation and motility in KIT-positive progenitors and tumor cells.

Taken together, these studies support a view in which RTKs form an interconnected scaffold that maintains CSC pools, links them to metabolic and epitranscriptomic programs, and modulates their response to microenvironmental cues and therapy, including radiotherapy and targeted agents. This complexity helps explain why RTK inhibitors can deplete CSC traits in preclinical models yet often show context‑dependent benefit in the clinic, and it motivates combinatorial strategies that target RTKs together with downstream pathways such as PI3K/AKT, MEK/ERK, or m^6^A regulators in tumors where RTK‑driven CSC dependence is viable.

#### JAK/STAT3 signaling pathway

JAK/STAT circuits, especially IL‑6/JAK2/STAT3 and IL‑6/JAK2/STAT5 modules, connect inflammatory cytokines to transcriptional programs that control CSC self‑renewal, phenotypic switching, immune escape and resistance to therapy in many tumor types. Elevated STAT3 activity is a recurrent feature of CSC‑like populations, and experimental STAT3 blockade often reduces tumorsphere growth, survival and metastatic traits. For example, an integrin‑linked kinase (ILK) to STAT3 pathway drives a mesenchymal, invasive GSC state and supports transition toward astrocyte‑like phenotypes that preserve tumor‑initiating capacity.^314^ Genetic or pharmacologic ILK inhibition lowers STAT3 phosphorylation, restricts invasive behavior and confines GSCs to a less plastic, progenitor‑like state in vitro and in mouse models. Other upstream inputs help maintain STAT3 activity in EGFR‑altered GSCs. Galectin‑1 (LGALS1) is highly expressed in EGFRvIII‑positive GSCs, that STAT3 binds the LGALS1 promoter, and that LGALS1 knockdown reduces neurosphere formation and tumor growth, placing Galectin‑1 as a component of an EGFRvIII-STAT3 network in this subset.^315^ A second kinase, BMX, is enriched in GSCs and couples receptor signaling to STAT3.^316^ The BTK/BMX inhibitor ibrutinib disrupts BMX‑STAT3 activation, depletes GSCs, lowers tumor burden and improves responses to radiotherapy while sparing normal neural progenitors.

Non‑coding RNA and cytoskeletal machinery reinforce IL‑6 to STAT3 signaling inside the stem‑like compartment in breast cancer models. The long non‑coding RNA XIST acts as a regulator of breast CSCs that carry ALDH‑positive epithelial and CD24^low^/CD44^high^ mesenchymal phenotypes.^317^ XIST knockdown reduces ALDH‑positive and CD24^low^/CD44^high^ fractions, impairs sphere formation and tumor initiation, and suppresses IL‑6 production from bulk tumor cells. Exogenous IL‑6 restores STAT3 phosphorylation and self‑renewal, and mechanistic work shows that XIST acts as a sponge for miR‑let‑7a‑2‑3p to de‑repress IL‑6 and drive STAT3‑dependent stem factors such as c‑MYC, KLF4 and SOX9.^317^ In TNBC, the ERM‑family protein moesin (MSN) forms a complex with phosphorylated STAT3 and amplifies an IL‑6 to MSN/STAT3 to LPAR1 cascade that supports mammosphere formation and Adriamycin resistance.^318^ Disruption of MSN or pharmacologic STAT3 inhibition with atovaquone breaks this loop, reduces stem‑like growth and restores chemosensitivity in preclinical models.

Colorectal cancer provides additional examples of STAT3‑centered CSC control. AKAP12, a scaffolding protein previously considered a tumor suppressor, is enriched in colorectal CSCs. AKAP12 loss diminishes colony and sphere formation, reduces CSC marker expression and lowers xenograft growth, whereas AKAP12 overexpression promotes these traits through an AKAP12 to PKC to STAT3 route.^319^ The transcription factor E2F3 is also upregulated in colon cancer spheres. Enforced E2F3 expression increases stem marker levels, migration and invasion, while E2F3 knockdown reduces these phenotypes and lowers STAT3 phosphorylation, and STAT3 inhibition blunts the E2F3‑driven stem program.^320^ Furthermore, a recent report reveals that IL‑6 uses distinct downstream routes to induce PD‑L1 depending on cell state: in non‑CSC colorectal cancer cells, IL‑6 activates a JAK to STAT3 to FRA1 axis that binds the PD‑L1 promoter, whereas in CSC‑enriched populations IL‑6 is redirected through SHP2 to PI3K to AKT, which increases ZEB1 and in turn drives PD‑L1 transcription.^321^ Dual targeting of STAT3 and PI3K combined with PD‑L1 blockade improves control of colorectal xenografts enriched for CSCs, illustrating how cytokine‑to‑STAT3 circuitry can rewire immune evasion in a state‑specific manner.

Related mechanisms appear in other epithelial tumors. In prostate cancer, the regulatory subunit of the condensin-2 complex, NCAPG2 is upregulated in aggressive disease and directly binds STAT3. NCAPG2 increases STAT3 occupancy at the MYC promoter, raises c‑MYC expression and correlates with CSC markers and tumorsphere formation.^322^ Genetic NCAPG2 knockdown reduces sphere growth, invasion and in vivo tumorigenicity, and these effects can be rescued by c‑MYC re‑expression, placing NCAPG2-STAT3-MYC as a driver of prostate CSC traits.^322^ JAK and STAT signals extend deeply into leukemic stem‑cell biology. In a Lmo2‑transgenic model of early T‑cell precursor acute lymphoblastic leukemia (ETP‑ALL), enforced STAT5 activation increases survival of chemoresistant pre‑leukemic stem cells and drives their evolution into full leukemic stem cells; genetic or pharmacologic STAT5 suppression slows disease progression and improves responses to cytotoxic therapy.^323^ A later study in AML shows that hematopoietic cytokines sustain leukemic stem‑like cells through partially distinct STAT modules: STAT5 together with ERK supports MCL1 expression, while STAT3 together with NF‑κB maintains BCL2.^324^ Combined inhibition of the STAT5 to MCL1 and STAT3 to BCL2 branches reduces colony formation and delays AML development in transplantation models.^324^

Taken together, these studies portray JAK-STAT networks as a shared scaffold for CSC maintenance across brain, breast, colorectal, prostate and hematologic malignancies. IL‑6, IL‑10 and matrix‑linked signals converge on STAT3 or STAT5 to coordinate self‑renewal, lineage plasticity, pro‑survival gene expression and immune escape, often through tumor‑specific adaptor nodes such as ILK, Galectin‑1, BMX, AKAP12 or NCAPG2. The most persuasive preclinical evidence supports strategies that lessen STAT3 activity in CSCs by blocking upstream kinases such as BMX, buffering cytokine inputs such as IL‑6 or IL‑10, or combining direct and indirect STAT3 inhibitors with chemotherapy, radiotherapy or PD‑1 or PD‑L1 blockade in tumors where JAK-STAT dependence of the CSC compartment can be monitored with suitable biomarkers.

### Mitochondrial dynamics and quality control in CSC persistence

CSCs seem to use mitochondrial flexibility as an internal adaptation system. It ties together metabolic state, redox balance, and organelle quality control.^325^ CSCs are not locked into one metabolic mode. Instead, they frequently switch between oxidative phosphorylation and glycolysis depending on context.^326^ This flexibility is closely tied to constant remodeling of mitochondrial networks through fusion, mediated by MFN1/2 and OPA1 and fission mediated by DRP1 along with outer-membrane adaptors such as FIS1, selective clearance via mitophagy, and new mitochondrial production through biogenesis.^327^ Fusion lets mitochondria share contents and buffer minor damage. It also supports efficient respiration and healthy cristae structure. Fission, on the other hand, helps isolate damaged segments for removal. It also allows rapid redistribution of mitochondria during asymmetric cell division and clonal expansion. Together, these opposing processes help CSCs maintain a balanced level of ROS.^325,328^ When ROS levels fall too low, stemness-associated signaling declines; when they rise too high, oxidative damage accumulates. The quality control pathways that execute this turnover include the ubiquitin-dependent PINK1/Parkin pathway and receptor-mediated mitophagy through BNIP3/NIX, which is especially active under stress and hypoxia. These programs are being recognized as contributors to drug resistance and metastatic ability in CSC-driven disease progression.^329^

MFN1/2-driven outer-membrane fusion paired with OPA1-driven inner-membrane fusion can create a mitochondrial architecture that supports sustained oxidative phosphorylation.^330,331^ This is particularly relevant in CSC states that favor oxidative metabolism. In NSCLC, a CSC-enriched population showed high OPA1 levels and increased mitochondrial fusion that supported stem-like properties. Mechanistically, enhanced lipogenesis induced OPA1 expression through the transcription factor SPDEF. Blocking lipogenesis or mitochondrial fusion pharmacologically reduced CSC markers and limited patient-derived organoid growth.^330^ This points to OPA1-driven fusion as a functional requirement for these cells. CSC persistence can also be reinforced by expanding the mitochondrial pool through biogenesis. In melanoma, CSC-enriched melanospheres had more mitochondria and higher levels of PGC1-alpha along with increased OXPHOS complex expression. These cells showed elevated membrane potential, oxygen consumption, ATP production, and ROS. Silencing PGC1-alpha or treating with pharmacologic inhibitors suppressed colony formation, spheroid growth, migration, invasion, and CSC marker enrichment.^332^ This links biogenesis-driven oxidative capacity to both stemness and aggressive behavior, at least in this cancer type. Together, these findings support a model where fusion and biogenesis programs act as a persistence mechanism for CSCs by maintaining high-quality, respiration-capable mitochondria under stress. However, the direction of these fusion effects can vary by cancer type and microenvironment.

On the fission and clearance side, DRP1-driven fragmentation and FIS1-linked mitophagy can serve as CSC maintenance mechanisms. They enable rapid mitochondrial turnover and damage containment. In glioblastoma, brain tumor-initiating cells (BTICs) had more fragmented mitochondria than non-BTIC tumor cells. BTICs showed activating DRP1 phosphorylation. Targeting DRP1 through RNA interference or pharmacologic inhibition triggered BTIC apoptosis and slowed tumor growth. DRP1 activity was linked to AMPK-mediated metabolic stress signaling, directly connecting DRP1-regulated fission to CSC-like survival under metabolic pressure.^333^ In AML, primitive LSCs depended on elevated FIS1 levels to drive the mitophagy needed for self-renewal and survival. Depleting FIS1 reduced mitophagy and triggered differentiation and cell-cycle arrest. AMPK acted upstream of this FIS1-mitophagy axis.^327^ Extending this to therapy resistance, prostate cancer stem-like cells (PCSCs) treated with the androgen receptor inhibitor enzalutamide showed mitophagy-dependent adaptation. CAMK1D promoted PCSC expansion and enzalutamide resistance by activating AMPK at Thr^140^ and engaging PINK1-dependent mitophagy. Targeting the CAMK1D/AMPK pathway depleted PCSCs and worked synergistically with enzalutamide in mouse models.^334^ Taken together, these studies across different cancer types support a framework where CSC persistence can be sustained through several routes: DRP1-centered fission linked to metabolic stress pathways, FIS1-supported mitochondrial turnover that maintains a self-renewing low-damage state, and therapy-induced recruitment of PINK1/Parkin mitophagy to tolerate mitochondrial injury. From a treatment perspective, these findings point toward combining standard therapies with mitochondrial quality control disruption. Examples include DRP1 pathway inhibition (such as Mdivi-1, tested in preclinical BTIC models), OPA1/fusion blockade (used to constrain NSCLC CSC pools), or mitophagy inhibition to prevent therapy-driven CSC enrichment. That said, normal stem and progenitor cells share many of the same mitochondrial quality control requirements, therefore, on-target toxicity remains a concern that must be carefully evaluated. Overall, the evidence shows that CSCs across multiple cancer types depend on tightly regulated mitochondrial dynamics to maintain their stem-like properties, drug resistance, and survival under stress, making these pathways promising yet challenging therapeutic targets.

## CSC-associated mechanisms of therapy resistance

CSCs are thought to contribute to therapy resistance and recurrence through self-renewal, differentiation into heterogeneous cancer-cell populations, and therapy-induced phenotypic switching. Resistance to one chemotherapeutic agent can often confer cross-resistance to other drugs,^12^ indicating that CSC-mediated resistance mechanisms and oncogenic cues should be considered when developing more potent treatment strategies and improving clinical outcomes.

### Intrinsic genetic and epigenetic factors driving resistance

**Fig. 5:**
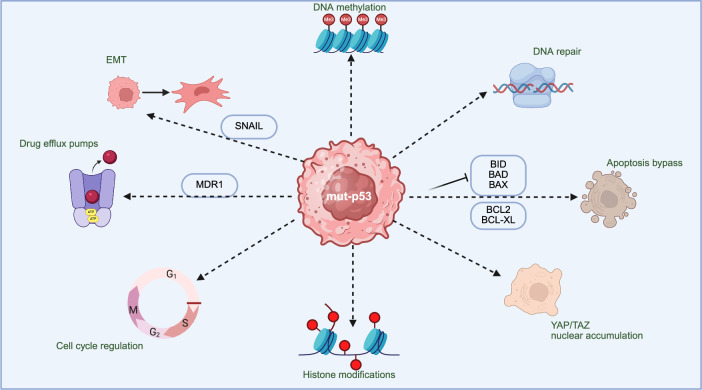
Mutant p53-centered intrinsic genetic and epigenetic mechanisms that can endow CSCs with therapy resistance. Loss-of-function mutations in tumor-suppressor genes and gain-of-function mutations in oncogenes reprogram CSC transcriptional profiles. In parallel, mutant or dysfunctional p53 can cooperate with oncogene activation, altered DNA and histone methylation, enhanced DNA-repair capacity, apoptosis evasion and overexpression of ATP-binding-cassette drug-efflux pumps to sustain therapy-resistant CSC states. Because these mechanisms vary across tumor types, the figure is intended as a conceptual framework rather than a universal pathway map. Created in BioRender. Uddin, K. (2026) https://BioRender.com/4b6v31o

CSCs harbor intrinsic features, including diverse genetic and epigenetic alterations, that strengthen their defenses against therapy and create conditions favorable to treatment resistance. These changes support evasion of drug effects while maintaining self-renewal and tumor-initiating capacity, thereby contributing to disease persistence and relapse. Genetic mutations within CSCs form one major axis of resistance, reshaping pathways that regulate DNA damage responses, cell cycle progression, and survival signaling.^1,10,335^ These mutations often activate oncogenes or inactivate tumor suppressor genes, disrupting pathways that maintain cellular homeostasis. For example, TP53 mutations, which affect the tumor suppressor p53, are common in CSC-enriched populations and have been linked to increased survival and proliferation across several cancer types.^336^ The p53 protein coordinates DNA repair, cell cycle arrest, and apoptosis, so mutations that impair its function permit continued proliferation and survival even during anticancer treatment. In parallel with these genetic changes, CSCs also undergo epigenetic alterations that contribute to therapy resistance. DNA methylation, histone modifications, and chromatin remodeling can shift gene expression without changing the underlying DNA sequence, reprogramming pathways that control differentiation, drug response, and stress adaptation.^1,337,338^ These changes can result in the silencing of genes that are required for regulating the cell cycle, apoptosis, and differentiation. For instance, hypermethylation of promoter regions of genes involved in cell cycle control and apoptosis can lead to their repression, enabling CSCs to bypass programmed cell death mechanisms and continue proliferating despite therapeutic interventions.^36,339^ The dynamic interplay between genetic and epigenetic alterations in CSCs emphasizes the complexity of their resistance mechanisms. For example, the aberrant methylation of DNA repair genes can render CSCs less susceptible to DNA-damaging agents, while the overexpression of drug efflux pumps, driven by mutations or epigenetic modifications, can decrease the intracellular concentration of chemotherapeutic drugs, thereby reducing their efficacy. Moreover, the plasticity of CSCs, partly driven by these intrinsic factors, allows them to adapt to and survive in varying microenvironments, further complicating the efforts to target them effectively. This plasticity, characterized by the ability of CSCs to switch between different phenotypic states, is influenced by both genetic and epigenetic changes and contributes to the heterogeneity observed within tumors.^340,341^ Addressing the challenge posed by the genetic and epigenetic foundations of CSC-induced resistance necessitates the development of innovative therapeutic strategies that can specifically target these alterations (Fig. 5).

### Avoidance of cell death and enhanced DNA repair in CSCs

Chemotherapeutic agents and ionizing radiation primarily exert their cytotoxic effects through the induction of DNA damage, which activates the DNA damage response (DDR). A failure to elicit an effective DDR can result in the accumulation of mutations and genomic instability, thereby leading to therapy resistance. While bulk cancer cells often exhibit impaired DNA repair mechanisms, CSCs have enhanced DNA damage repair capacity compared to their non-stem counterparts, supporting them to survive genotoxic stress and evade therapy-induced cell death.^342^

Generally, CSCs resist DNA damage-induced cell death through multiple mechanisms, including the upregulation of DNA repair pathways and activation of anti-apoptotic signaling cascades. For instance, in glioma cells, CD133^+^ CSCs exhibit more robust activation of DNA damage checkpoints in response to radiation, thereby demonstrating superior repair of the radiation-induced DNA damage relative to CD133^-^ tumor cells.^343,344^ Mechanistically, DNA damage triggers the ATM and ATR kinases, which form complexes with PARP-1 and BRCA1, resulting in the phosphorylation of checkpoint kinases CHK1 and CHK2.^344^ This leads to the activation of target proteins and induction of DNA repair capacity. In contrast, pharmacological inhibition of CHK1 and CHK2 sensitizes CSCs to radiotherapy, suggesting the potential of targeting DDR components in stem-like cancer cells.^12,345^ In breast cancer models, inhibiting the ATR-Chk1 axis has been shown to reverse radiotherapy resistance. In parallel, CSCs exploit survival-promoting pathways to evade apoptosis. For example, activation of the Rho/Rho-associated protein kinase (ROCK) signaling axis promotes survivin expression, thereby protecting CSCs from therapy-induced apoptotic cell death and enabling resistance to cytotoxic treatments.^71^ Emerging evidence also suggests ferroptosis as a promising strategy to overcome CSC-associated resistance. Induction of ferroptosis has been demonstrated to sensitize CSC populations to chemotherapy and mitigate radioresistance driven by augmented DNA repair capacity.^12^

### ROS-mediated radiation and chemotherapeutic resistance

Reactive oxygen species (ROS) are oxygen-containing signaling molecules characterized by highly reactive properties, and are linked to several physiological processes, including respiration, energy consumption, and enzyme activities. ROS encompass both radical species (e.g. superoxide anion, O_2_^-^) and non-radical derivatives (e.g., hydrogen peroxide, H_2_O_2_) and are tightly regulated under homeostatic conditions.^346^ CSCs maintain intrinsically lower basal ROS levels and augmented antioxidant defenses compared to their non-stem counterparts. This redox profile contributes to their increased survival, maintenance of stemness, and resistance to oxidative stress, particularly in response to radiotherapy.^328^ In parallel, excessive ROS production during chemotherapy induces oxidative stress in CSCs, leading to functional impairment and increased cell death.^165,347^ Additionally, oxidative stress can trigger phenotypic plasticity within the CSCs, promoting the transition from quiescent, ROS-low mesenchymal-like CSCs to proliferative, ROS-high epithelial-like CSCs, a shift related to enhanced tumorigenic potential. In CSCs, low ROS levels support the maintenance of stem cell quiescence/dormancy, an adaptive state that underlies therapeutic evasion.^348^ Redox regulation within the CSC population is integral to cancer recurrence, modulation of cellular signaling, and the development of therapy resistance.^346^

ROS are also typically involved in inducing DNA damage and triggering the DNA damage response. The efficacy of many anticancer therapeutics is based on their ability to increase ROS levels in cancer cells. Despite this, CSCs display resistance to such strategies due to their elevated redox buffering capacity and efficient DNA repair mechanisms. For example, Forkhead box protein M1 (FoxM1) has been shown to downregulate intracellular ROS levels, thereby supporting CSC-associated stemness and drug resistance.^347^ Similarly, high-energy radiation therapy can also generate ROS and trigger extensive DNA damage. However, CSCs remain largely refractory to radiation-induced cytotoxicity, leading to radioresistance, tumorigenesis, and tumor recurrence.^178,349^ Supporting this, it has been demonstrated that radiation can induce MSI1 expression, enhancing tumor invasion through VCAM1, which is associated with increased glioblastoma radioresistance.^350^ The redox regulation in CSCs is further governed by transcriptional and enzymatic regulatory networks, including activation of Nrf2 and overexpression of peroxiredoxin-2 (Prx2).^351,352^ These factors collectively bolster the antioxidant capacity of CSCs and contribute to chemoresistance in several cancer types.^165,347^ Targeting these redox routes may represent therapeutic opportunities. For example, Nrf2 silencing has been shown to retain high levels of ROS and reverse chemoresistance in CD44-enriched CSC populations.^12^ Moreover, Thy1^+^CD24^+^Lin^-^ CSCs are relatively more radioresistant compared with non-CSC population, underlining the importance of redox homeostasis in CSC-mediated therapeutic evasion.^328^

### ALDH-mediated multidrug resistance

Multidrug resistance (MDR) refers to the ability of cancer cells to withstand various anti-cancer therapies with distinct chemical structures and modes of action. MDR is a major obstacle to effective cancer therapy and contributes to cancer-related deaths among patients receiving either conventional chemotherapeutics or targeted drugs. The underlying mechanisms of MDR are multifaceted and include enhanced metabolism and efflux, deregulated growth factor signaling, increased DNA repair capacity, and a variety of genetic and epigenetic alterations, such as gene mutations, amplifications, and chromatin remodeling.^12,180,353,354^ Within this context, CSCs display a heightened capacity for MDR, which is closely associated with the expression of ALDH enzymes, particularly ALDH1A1. CSCs expressing ALDH1A1 exhibit significantly greater resistance to the EGFR tyrosine kinase inhibitor gefitinib and other chemotherapy drugs including cisplatin, etoposide, and fluorouracil, when compared to ALDH1A1-negative cells.^343,355–357^ Mechanistically, ALDH can mediate MDR via multiple pathways, including the maintenance of low intracellular ROS levels which protect cells from oxidative stress-induced apoptosis, and the detoxification of cytotoxic aldehydes, both of which enhance cell survival in the presence of chemotherapeutic stress.^165^ Typically, cytosolic ALDHs oxidize toxic intracellular aldehydes into less harmful carboxylic acids. For instance, in leukemia cells, ALDHs specifically detoxify aldophosphamide in the CSC subpopulation, conferring chemoresistance to alkylating agents such as cyclophosphamide.^358^ In addition to ALDH-related mechanisms, ABC transporter proteins are prominently overexpressed in CSCs and function as critical mediators of drug efflux, limiting intracellular drug accumulation and contributing to CSC-associated MDR.^12,71^ An alternative model for CSC-mediated drug resistance posits that the side population (SP) cell fraction is enriched for CSCs.^12^ This subset displays increased expression of drug transporter proteins including MDR1/ABCB1, BCRP/ABCG2, and MRP1/ABCC1, which actively efflux cytotoxic drugs, thereby contributing to resistance to chemotherapeutic compounds.^343^

### EMT and CSC markers in therapy resistance

The EMT is a reversible process in which epithelial cells undergo phenotypic reprogramming characterized by the loss of apical-basal polarity and intercellular junctions, accompanied by the acquisition of mesenchymal characteristics such as enhanced migratory capacity, invasiveness, and resistance to apoptosis.^359^ EMT plays an important role in various physiological and pathological contexts, including embryonic development, wound healing, tissue fibrosis, cancer progression, migration, tumor stemness, and therapeutic resistance. Notably, EMT activation is strongly associated with the development of drug resistance, largely through its role in fostering the enrichment and sustained presence of CSC phenotypes. Conventional anticancer therapies often fail to eradicate CSCs due to EMT-driven phenotypic plasticity, leading to CSC-mediated therapy resistance and cancer recurrence. For example, EMT-associated transcription factor Snail induces AXL receptor tyrosine kinase, whose ligand-dependent activation by GAS6 facilitates resistance to EGFR blockade by erlotinib, in Snail-expressing cancer cells.^360–362^ Similarly, Snail or Twist mediate gemcitabine resistance in pancreatic cancer cells through the modulation of drug-inactivating enzymes and efflux transporters.^363^

Building on these molecular insights, emerging evidence further supports a broader role of EMT in shaping the therapy-resistant tumor phenotype. In particular, analyses of chemotherapy responses in breast cancer patients have revealed a strong correlation between therapeutic resistance and increased expression of stromal-cell-associated genes, a transcriptional profile likely driven by activation of EMT within cancer cells.^364,365^ This may reflect the contribution of EMT-linked transcriptional reprogramming to the tumor’s ability to adapt to and withstand therapeutic stress. Moreover, EMT enables tumor cells to engage in bidirectional crosstalk with the surrounding tumor microenvironment, including stromal and immune cells, which can further support therapy resistance. For example, EMT can promote immune evasion by upregulating checkpoint molecules, thereby attenuating cytotoxic T cell responses and reducing immunotherapy efficacy.^363,366,367^

In diverse cancer types, the activation of EMT is closely related to the acquisition of CSC characteristics and it is predominantly confined to CSC-enriched subpopulations.^9^ A key early step in CSC-mediated metastasis involves the acquisition of invasive traits through EMT, which also contributes to therapy resistance.^12^ EMT activation functionally reinforces the CSC phenotype by promoting invasive capabilities and driving chemoresistance, primarily through extensive reprogramming of the tumor microenvironment.^359^ One mechanistic link between EMT and the CSC phenotype involves the FBXW7-ZEB2 axis, which integrates EMT signaling with tumor microenvironmental remodeling, resulting in suppressed apoptosis, expansion of colorectal CSCs, and increased chemotherapy resistance.^368^ EMT not only facilitates metastatic dissemination but also promotes the expansion of self-renewing cells, thereby sustaining the CSC pool. Notably, signaling pathways that are activated during EMT are strikingly comparable to those that drive CSCs, such as WNT/β-catenin, TGF-β/SMADs, and other signaling pathways required for CSC self-renewal and maintenance.^363^ Among EMT-associated transcriptional regulators, ZEB1 has emerged as a key player in inducing stemness and chemoresistance through modulation of MGMT expression in malignant glioma.^343^ Similarly, metadherin has been shown to indirectly activate Twist1 expression by facilitating histone H3 acetylation, thereby enhancing CSC traits and drug resistance.^12^ Given these findings, EMT activation serves not only as a mechanistic driver of CSC function but also as a predictive marker for anticipating the emergence of chemotherapy resistance during cancer treatment.^359^

Several CSC-associated markers have been directly implicated in stemness and therapy resistance, highlighting their potential utility as prognostic indicators and therapeutic targets. For example, CD133 (Prominin-1) is a well-established stemness marker linked to drug resistance in multiple cancers. In lung cancer, CD133-enriched CSCs display increased expression of the drug efflux transporter ABCG2, resulting in resistance to platinum- and taxane-based chemotherapies. Moreover, low-dose platinum exposure induces DNA damage that further enhances ABCG2 expression, and expands the CD133^+^ population, promoting multidrug resistance.^71,343^ Similarly, CD44, particularly its variant isoform CD44v, is a CSC biomarker and a key regulator of cancer aggressiveness, metastasis, and chemoresistance. In colon cancer, CD44 variant (CD44v6) expression contributes to resistance against fluorouracil, oxaliplatin and leucovorin (FOLFOX),^369^ while in osteosarcoma, CD44 promotes doxorubicin resistance through the regulation of MDR1 protein.^71^ Other cell surface markers associated with CSC-mediated resistance include CD166, CD49f, CD24, and CD9. CD166, also known as ALCAM, is enriched in CSCs from colon, stomach, ovarian, and head and neck cancers,^71,370^ and in ovarian cancer it has been shown to exhibit enhanced cell migration and adhesion abilities, tumorigenicity, and CSC-associated resistance to conventional anticancer drugs.^371^ CD49f (integrin α6) is also associated with metastasis, radioresistance and taxane resistance;^372^ in TNBC, docetaxel correlates with the expansion of CD49f+ population.^373^ Another prognostic CSC marker linked to chemoresistance, CD24, facilitates a CSC phenotype and therapy resistance by regulating miRNAs and promoting a quiescence-like state in ovarian cancer.^374^ CD9, a tetraspanin protein, has been implicated as a new biomarker of AML stem cells.^375^ Notably, CD9 blockade has been demonstrated to suppress disease progression of high-risk pediatric B-cell precursor acute lymphoblastic leukemia and enhance chemosensitivity.^376^ Specifically, p38 MAPK regulates the expression of ALDH isoforms, such as ALDH2, conferring therapy resistance.^358^ ALDH1^+^ CSCs are particularly resistant across several malignancies; for instance, in ovarian cancer, ALDH1 expression is correlated with platinum resistance by regulating cell cycle checkpoints and DNA repair, ultimately leading to clinical relapse.^71^

### CSC dormancy-mediated radiation and chemotherapy resistance

Cellular dormancy, or quiescence, is a reversible state in which cells exit the active cell cycle and enter the non-dividing G_0_ phase. Despite this arrest, they retain the potential for cell division in response to mitotic stimuli. A subpopulation of CSCs has been identified in this slow-cycling, quiescent state, enabling them to evade cytotoxic effects of chemotherapy and contribute to minimal residual disease.^165^ CSCs often remain quiescent in response to different microenvironmental stresses including hypoxia and nutritional deprivation, but can regain their characteristics under favorable physiological conditions, thereby fueling drug resistance, cancer relapse, and metastases.^71^ However, the dormant tumor cells comprise both CSC and non-CSC populations. Given that most chemotherapeutic and radiotherapeutic strategies are designed to eliminate actively proliferating cells, dormancy confers a significant survival advantage to CSCs, often resulting in tumor relapse. The regulation of CSC dormancy is governed by a network of intracellular signaling pathways and extrinsic cues. Key regulators include tumor suppressors, CDKs, the Notch signaling pathway, epigenetic modifications, and components of the tumor microenvironment such as niche-secreted factors, and immune cells. For example, p38-mediated MAPK1 activation has been implicated in the induction of CSC dormancy and the promotion of therapy resistance in cancer.^358,377^ Conversely, activation or suppression of developmental pathways can influence dormant-state entry and exit, although the evidence is context-dependent.^83,348,378,379^ In colorectal cancer, chemotherapy enriches a pre-existing population of ZEB2⁺ quiescent CSCs that express elevated levels of pCRAF and pASK1; ZEB2 overexpression upregulates this axis, promoting quiescence, stemness/EMT traits, and chemoresistance, resulting in a chemotherapy-unresponsive state.^380^ Similarly, in bladder cancer, it has been reported that quiescent CSCs re-enter the cell cycle in response to gemcitabine and cisplatin treatment, highlighting their plasticity and adaptability.^165^ Despite the lack of well-defined surface markers, quiescent CSCs possess unique features that can aid in their identification. These include label-retention, reduced RNA content, and the absence of proliferative markers.^381–383^ A deeper understanding of these characteristics, along with the molecular mechanisms underlying dormancy may help to develop effective therapeutic strategies targeting the elusive quiescent CSC population.

### Niche-mediated resistance

**Fig. 6:**
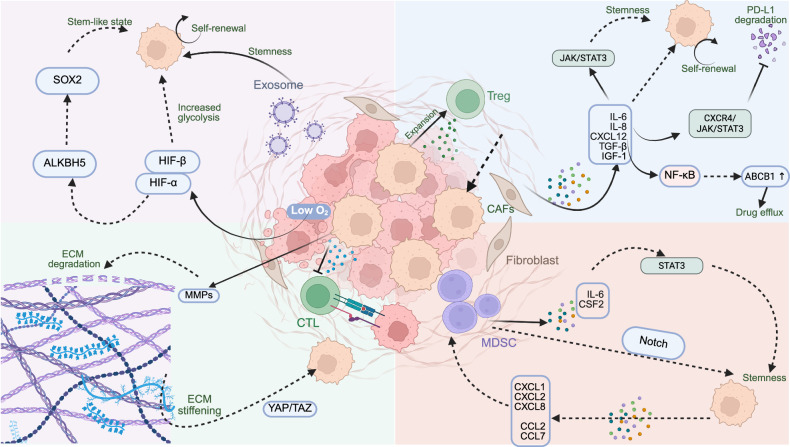
Crosstalk between CSC niches and the tumor microenvironment in therapy resistance. In hypoxic and stiff ECM regions, HIF-α/HIF-β, ALKBH5, SOX2, MMPs and YAP/TAZ support stem-like states, ECM remodeling and self-renewal. CSC-derived exosomes and CAF-secreted factors such as IL-6, IL-8, CXCL12, TGF-β and IGF-1 can activate JAK/STAT3, NF-κB, Notch and ABC transporter programs that reinforce stemness and drug efflux. Immune interactions include suppression of CTL activity, expansion of Treg cells, and recruitment of myeloid-derived suppressor cells (MDSCs). The upper-right module depicts CXCR4/JAK/STAT3-linked inhibition of PD-L1 degradation, a mechanism that can stabilize PD-L1 and contribute to PD-1-mediated T-cell exhaustion in selected contexts. Created in BioRender. Uddin, K. (2026) https://BioRender.com/820awzy

CSCs reside in a specialized and dynamic microenvironment known as the CSC niche, which sustains their stem-like properties, supports survival under stress, and enables therapy resistance and tumor relapse. This niche functions not as a passive scaffold but as an active, adaptive ecosystem where CSCs both receive and transmit molecular cues that remodel the tumor milieu and reinforce intratumoral hierarchies (Fig. 6). The resulting microenvironmental influences facilitate a sanctuary for CSCs, enabling them to evade therapeutic assaults and contribute significantly to tumor progression and recurrence.^384^

The extracellular matrix and its associated proteins can regulate CSC properties by providing physical and biochemical niches enhancing their capacity to initiate tumors.^10^ Beyond these structural cues, metabolic and oxygen-sensing pathways within the niche play pivotal roles in dictating CSC fate. Under hypoxic conditions, the stabilization of HIFs, particularly HIF-1α and HIF-2α, activates a multitude of survival pathways that bolster CSC resistance to therapy.^385–387^ By activating glycolysis and other metabolic pathways, hypoxia ensures an energy supply that supports CSC survival, proliferation, and maintenance, even in the hostile environment of a growing tumor. Furthermore, hypoxia can induce the expression of genes associated with drug resistance, angiogenesis, and metastasis in CSCs. This not only promotes CSC survival in situ but also their capacity to seed new tumors at distant sites, contributing to the spread of cancer within the body. This molecular control extends to epigenetic regulation; for instance, in endometrial CSCs (ECSCs), hypoxia and HIF-1α increase the expression of the m^6^A demethylase ALKBH5, which promotes SOX2 transcription via mRNA demethylation, thereby maintaining the ECSC stem-like state and tumorigenicity.^388^ The combined effects of niche interactions and hypoxia highlight the complexity of targeting CSCs within the tumor microenvironment. These factors emphasize the necessity for novel therapeutic strategies that can disrupt the protective niche of CSCs, overcome the adaptive responses driven by hypoxia, and ultimately target the CSC population more effectively. Addressing these challenges requires a multifaceted approach, integrating targeted therapies against CSCs with strategies aimed at modifying the tumor microenvironment to render it less hospitable to CSC maintenance and survival.

A defining feature of the CSC niche is the complex, bidirectional communication mediated by extracellular vesicles (EVs) and exosomes, which exchange proteins, RNAs, and lipids between CSCs, non-stem cancer cells (NSCCs), and stromal components. In pancreatic ductal adenocarcinoma (PDAC), CSCs orchestrate a structured communication network, termed the EVNet, preferentially directing EV trafficking toward NSCCs.^389^ This CSC-led signaling hierarchy enhances tumor adaptability and growth through the agrin/LRP4/YAP axis. Agrin-enriched EVs from CSCs activate YAP signaling in recipient cells, promoting proliferation and conferring aggressive phenotypes. Blocking this CSC-to-NSCC communication suppresses tumor growth, highlighting CSCs as key regulators of intratumoral networks.^389^

Similar EV-mediated mechanisms drive resistance in other cancers. Hypoxia-induced glioma stem cells (H-GSCs) transmit oncogenic signals via exosomes carrying lncRNAs and miRNAs. Linc01060-containing exosomes, for instance, activate the MZF1/c-Myc/HIF-1α feedback loop in glioma cells, enhancing glycolytic metabolism and sustaining malignancy.^390^ Simultaneously, EV-encapsulated miR-30b-3p from H-GSCs promotes temozolomide resistance by repressing the apoptotic regulator RHOB.^391^ These examples demonstrate that stress conditions not only select for CSC phenotypes but also reprogram their EV cargo to disseminate pro-survival and resistance signals across the tumor mass. Similarly, environmental acidity exerts a selective stress response, fostering CSC enrichment, metabolic reprogramming, and promoting the emergence of aggressive subpopulations exhibiting increased ALDH activity and elevated β-catenin signaling.^392^

The CSC niche is reinforced through reciprocal interactions with stromal and immune cells, which collectively create a protective barrier against therapy. Cancer-associated fibroblasts (CAFs) shape a pro-stemness microenvironment through metabolic and paracrine mechanisms. In intrahepatic cholangiocarcinoma (ICC), CAFs instruct MDSCs to upregulate leukotriene B4 (LTB4) synthesis via the 5-lipoxygenase pathway, which activates BLT2 receptors on CSCs to enhance stemness and chemoresistance.^393^ This CAFs/MDSCs/CSC metabolic circuit, driven by IL-6/IL-33 and lipid mediators, exemplifies how stromal subsets cooperate to optimize CSC maintenance.

Within the immune compartment, tumor-associated macrophages (TAMs) act as major architects of the CSC niche across tumor types. In prostate cancer, CCL5 secreted by TAMs promotes CSC self-renewal and metastatic dissemination through β-catenin/STAT3 activation.^394^ In TNBC, CCL2/Akt/β-catenin signaling downstream of M2-like TAMs drives both EMT and CSC enrichment.^395^ Interestingly, in oral squamous cell carcinoma (OSCC), M1-like TAMs can paradoxically enhance stemness through an IL-6/STAT3/THBS1 feedback loop, suggesting the context-dependent plasticity of macrophage-CSC crosstalk within different tumor contexts.^396^ CSCs also promote immune evasion by modulating immune cells through EVs. In esophageal carcinoma, ALDH⁺ CSC-derived exosomes transfer O-GlcNAc transferase (OGT) to CD8⁺ T cells, elevating PD-1 expression and impairing cytotoxic responses, thereby protecting CSCs from immune-mediated elimination.^397^

Taken together, these studies portray the CSC niche as a multilayered communication hub integrating vesicular signaling, metabolic adaptation, and immune modulation. Rather than functioning as a static reservoir, the CSC niche dynamically evolves through reciprocal interactions between CSCs and their surroundings. Elucidating these cooperative and context-specific networks will be critical for developing therapeutic strategies that disrupt CSC-directed crosstalk, thereby dismantling the self-sustaining ecosystem that fuels tumor progression and relapse.

### Signaling pathways of therapy resistance in CSCs

**Fig. 7:**
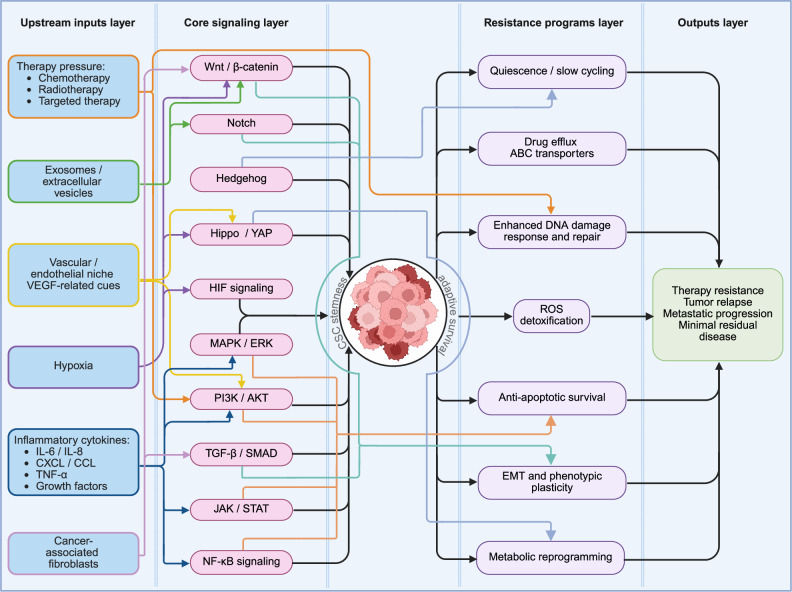
Mechanisms underlying cancer stem cell-mediated therapy resistance. Therapy pressure from chemotherapy, radiotherapy and targeted therapy, together with tumor microenvironment-derived cues including exosomes/extracellular vesicles, vascular or endothelial niche signals, hypoxia, inflammatory cytokines and cancer-associated fibroblasts, converge on a core signaling network that sustains CSC stemness and adaptive survival. These pathways include WNT/β-catenin, Notch, Hedgehog, Hippo/YAP, HIF, MAPK/ERK, PI3K/AKT, TGF-β/SMAD, JAK/STAT and NF-κB signaling, all of which have been implicated in the maintenance of CSC phenotypes, cellular plasticity, survival under therapeutic stress and resistance to anticancer treatment. These signaling pathways support resistance programs including quiescence/slow cycling, ABC-transporter-mediated drug efflux, enhanced DNA damage response and repair, reactive oxygen species detoxification, anti-apoptotic survival, EMT-associated phenotypic plasticity and metabolic reprogramming, including mitochondrial dynamics and mitophagy. Collectively, these adaptive programs enable CSCs to survive treatment, persist as residual disease, and drive therapy resistance, tumor relapse and metastatic progression. Created in BioRender. Uddin, K. (2026) https://BioRender.com/o0g6nhy

Several signaling pathways that regulate CSC behavior also shape how these cells respond to therapy. Among these, Notch, WNT/β-catenin, and Hh signaling can preserve CSC-associated programs under therapeutic stress, promote survival, and facilitate recurrence (Fig. 7). Having discussed their roles in CSC maintenance earlier, this section focuses on how these pathways and closely associated networks such as ABC transporters and PI3K/Akt/mTOR signaling contribute to therapy resistance.

Notch signaling is a key determinant of drug-tolerant CSC-like states. In breast cancer models, Notch activation has been associated with a drug-resistant phenotype, and Notch receptor/ligand expression can increase following exposure to agents such as doxorubicin, docetaxel, or endocrine therapies.^398^ Notch signaling can also promote EMT programs, which are frequently linked to chemoresistance and metastatic traits.^399^ Furthermore, upregulation of Notch3 in ovarian cancer has been associated with increased platinum resistance and expansion of CSC fractions.^400^ In line with these findings, pharmacologic or genetic Notch inhibition can sensitize CSC-enriched populations to chemo- and radiotherapy, reducing clonogenic survival and partially reversing CSC-associated resistance across multiple models.

The WNT/β-catenin signaling has been linked to therapy resistance across tumor contexts.^12,58,180^ In pancreatic cancer models, WNT7B can drive autocrine β-catenin signaling and anchorage-independent growth.^401^ In neuroblastoma, chemoresistant subpopulations show increased expression of the WNT receptor FZD1, which promotes β-catenin nuclear accumulation and activates resistance-associated transcriptional programs, including MDR1/ABCB1.^343^ Collectively, these findings position WNT/β-catenin as a hub connecting stemness programs and therapy resistance.

Aberrant activation of the Hh pathway also contributes to therapy resistance by sustaining CSC survival after cytotoxic stress and promoting tumor regrowth following chemo- or radiotherapy, often through dysregulation of core components such as PTCH1, SMO, SUFU, and GLI transcription factors.^180,402^ A prominent resistance mechanism involves GLI-dependent induction of ABC transporters, which increases drug efflux and lowers intracellular drug accumulation. Transporters reported downstream of Hh signaling include MDR1/ABCB1, BCRP/ABCG2, and MRP1/ABCC1.^403^ Beyond drug efflux, Hh signaling has been linked to broader resistance-associated networks, including cytokine signaling (e.g., IL‑6), regulatory microRNAs (e.g., miR‑200 family members), and long non-coding RNAs, which together can contribute to resistance to platinum-based chemotherapy and targeted therapies (including EGFR TKIs).^12^ Accordingly, inhibition of Hh signaling has been reported to downregulate stemness markers and restore chemosensitivity in multiple tumor models.^403^ For example, blockade of Shh pathway activity can reverse resistance to 5‑FU and platinum-based agents in colorectal CSC models and has also been described in CSC-enriched gastric cancer and leukemic populations in vitro and in xenografts.^12,71^

ABC transporters constitute a critical downstream effector system that integrates signals from Notch, WNT, Hh, and other stemness-related pathways. Overexpression of MDR1/ABCB1, BCRP/ABCG2, and MRPs/ABCCs in CSC-enriched fractions can actively efflux diverse chemotherapeutic agents, limiting intracellular drug exposure and reducing efficacy.^403–405^ MDR1/ABCB1 has been implicated in reduced sensitivity to taxanes and anthracyclines used in breast and ovarian cancer, supporting survival of MDR1-high, therapy-tolerant subpopulations and promoting recurrence.^406^ MRPs, particularly MRP1/ABCC1, have also been associated with resistance to platinum compounds and alkylating agents. For example, MRP1 overexpression in lung cancer models has been connected to diminished cisplatin sensitivity.^405^ These observations underpin continued efforts to inhibit ABC transporters pharmacologically and design agents that evade efflux, although clinical translation has been challenging.

CSCs also exploit the PI3K/Akt/mTOR axis, a central survival and growth pathway that is frequently hyperactivated in resistant tumors. In glioblastoma, CSC-enriched populations often show elevated PI3K/Akt/mTOR activity, and pathway inhibition can impair self-renewal and sensitize tumors to chemo- and radiotherapy in preclinical studies.^407,408^ In breast cancer, CD44⁺/CD24⁻ CSC-enriched fractions similarly exhibit increased PI3K/Akt/mTOR signaling, and inhibition with PI3K or mTOR inhibitors has been reported to reduce CSC frequency and tumor burden in model systems.^409^ Colorectal CSCs likewise engage this pathway to suppress apoptosis and sustain proliferation in the presence of cytotoxic stress, and targeting nodes within the pathway can deplete CSC-enriched pools and reduce recurrence in experimental settings. Importantly, PI3K/Akt/mTOR signaling is extensively interconnected with WNT/β-catenin, Hh, and other stemness-associated networks,^194^ creating a robust, partially redundant resistance circuitry.

Collectively, these data indicate that developmentally conserved pathways and their downstream effector systems not only sustain CSC-like states but also orchestrate multilayered mechanisms of therapy resistance. Targeting WNT, Hh, Notch, ABC transporters, and PI3K/Akt/mTOR therefore remains an attractive strategy to improve CSC eradication and long-term outcomes.^10,338^ However, the need to preserve normal stem-cell function, the risk of pathway compensation, and the extensive crosstalk among these networks highlight the importance of rationally designed combination strategies, biomarker-guided patient selection, and careful clinical translation.

## Challenges in targeting cancer stem cells for therapy

Addressing the challenges posed by CSCs in cancer therapy is a formidable task, largely due to the unique attributes of CSCs, their intricate relationship with the tumor microenvironment, and the obstacles in accurately identifying and effectively targeting them. These challenges are compounded by the heterogeneity and plasticity inherent to CSCs, which allow them to adapt and survive even in the face of aggressive treatments.

### Heterogeneity and plasticity of CSCs

CSCs within a single tumor display a remarkable level of heterogeneity, possessing diverse genetic and phenotypic profiles. This intra-tumor heterogeneity significantly complicates therapeutic strategies, as different CSC populations within the same tumor may exhibit varied responses to treatment. This variability leads to the incomplete eradication of CSCs, often resulting in tumor relapse. The diversity among CSCs within a tumor implies that a single therapeutic strategy is unlikely to be universally effective, necessitating the development of multi-targeted or combination therapies. This dynamic nature enables CSCs to adapt to environmental pressures, including therapeutic interventions, making them particularly resilient to treatments designed to target more static cellular populations. These patterns are supported by single-cell profiling, lineage tracing and clonal-barcoding studies that connect heterogeneous cell states with treatment selection and relapse dynamics.^56,106,107,132–134^

### Diagnostic and therapeutic implications

Identifying and isolating CSCs poses significant challenges, largely attributable to their rarity within tumors and the absence of specific, universally accepted markers. The diversity among CSCs implies that markers effective for one cancer type may not be universally applicable. A cancer-selective compilation of CSC markers is provided in Table 1, yet these markers require further validation. This underlines the need for an advanced understanding of CSC biology to improve identification methods.^70^ Present therapeutic strategies aimed at CSCs include the inhibition of pathways associated with CSC maintenance, targeting of CSC-supportive niches, and immunotherapy designed to eliminate CSC-enriched populations. One strategy in CSC-targeted therapy is blockade of signaling pathways linked to EMT and therapy resistance. For example, TGF-β inhibition has shown biologically relevant activity in early clinical studies, but the evidence should be interpreted cautiously. In a small randomized study of radiotherapy plus fresolimumab in chemoresistant metastatic breast cancer, the higher-dose arm showed stronger immune correlates and longer median overall survival than the lower-dose arm, but objective responses were not observed; these findings are hypothesis-generating rather than practice-changing.^410,411^ Similarly, targeting the HGF-MET signaling pathway, which has roles in cancer development and CSC regulation, has shown preclinical and early clinical promise for modulating tumor progression and drug resistance, but patient selection and biomarker validation remain essential.^412,413^

**Table 1:** Commonly used CSC-enrichment markers across selected tumor types

Cancer type	Commonly used CSC-enrichment markers	Supporting markers	Notes and references
Breast cancer	CD44^+^/CD24^−/low^, ALDH1^+^	EpCAM, CD49f, CD29, CD133, HER2	The CD44^+^/CD24^-/low^ phenotype is the canonical breast CSC population first validated by xenotransplantation, while ALDH1 identifies an overlapping but functionally important tumor-initiating subset.^7,10,31,75,84,265,372,373,464–466^
Glioblastoma	CD133, together with Nestin/SOX2	CD15 (SSEA-1), A2B5, CD44, L1CAM	CD133 remains the historical benchmark for glioblastoma stem-like cell enrichment, but current literature supports pairing it with Nestin and SOX2 to reflect neural stem-like identity. CD15/SSEA-1 and A2B5 are useful because they capture CD133-negative tumor-initiating populations and therefore better reflect glioblastoma heterogeneity.^10,89,258,467–470^
Lung cancer (mainly NSCLC)	CD133, CD44, and ALDH1	EpCAM, ABCG2, SOX2, OCT4, NANOG	In lung cancer, especially NSCLC, CD133, CD44, and ALDH1 are the most repeatedly used enrichment markers for tumor-initiating and therapy-resistant subsets. Additional markers such as EpCAM and stemness-associated transcription factors are useful, but they are better presented as supporting rather than primary markers.^10,355,356,471–473^
Colorectal cancer	LGR5, CD44, and CD133	EpCAM, CD166, CD24	LGR5 is the most lineage-informed colorectal stemness marker and is strongly linked to WNT-driven intestinal stem-cell identity, whereas EpCAM^high^/CD44^+^ and CD133^+^ populations remain foundational colorectal CSC phenotypes. CD166 can be added as a refinement marker when greater specificity is needed.^10,45,94,96,474^
Pancreatic ductal adenocarcinoma	CD44^+^/CD24^+^/EpCAM^+^ and CD133^+^/CXCR4^+^	c-MET, CD44v6, ALDH1, DCLK1	The CD44^+^/CD24^+^/EpCAM^+^ population is the classic tumor-initiating pancreatic CSC compartment, whereas CD133^+^/CXCR4^+^ marks a particularly invasive and metastatic subset. Presenting both marker combinations is a strong way to summarize pancreatic CSC biology without oversimplification.^58,87,404,475,476^
Hepatocellular carcinoma	EpCAM, CD24, and CD13	ICAM-1, CD90, CD133, CD44	In HCC, EpCAM is one of the most established liver CSC markers and is closely linked to hepatic progenitor-like programs. CD24 and CD13 enrich additional stem-like, chemoresistant, or quiescent CSC pools, while ICAM-1 is a useful functional adjunct marker for tumor initiation and dissemination. This combination provides a balanced and current summary for liver CSCs.^88,477–480^
Prostate cancer	CD44, CD133, ALDH1, α2β1 integrin	CD117, CXCR4, EpCAM, EZH2	Prostate CSC-enriched subsets are often AR^low/-^ and are studied in relation to therapy resistance and castration-resistant disease. CD44, CD133, ALDH activity, and α2β1 integrin have been used as enrichment markers, but functional validation remains necessary.^481–485^
Ovarian cancer	CD133, CD44, ALDH1, EpCAM	KIT, CD24, SOX2, Nestin	ALDH1^+^, CD133^+^, CD44^+^, and EpCAM^+^ populations are frequently used to enrich chemoresistant or tumor-initiating ovarian cancer cells, although marker combinations require tumor-context-specific functional validation.^486–488^

CSC markers are context-dependent enrichment tools rather than universal gold-standard identifiers. Marker combinations should be interpreted together with expression thresholds, isoforms, tumor context, and functional assays such as limiting-dilution transplantation or serial propagation

The hypoxic conditions within the tumor microenvironment also pose additional challenges.^232^ Targeting hypoxia-related factors like HIF-1α has been explored as a strategy to disrupt cancer cell glucose metabolism, offering a potential approach for CSC-targeted therapy.^414,415^ The therapeutic landscape is further complicated by the redundancy and interaction of signaling pathways within CSCs, such as the Notch, WNT, and Hedgehog pathways, potentially diminishing the efficacy of targeted therapies. Nevertheless, clinical and translational studies have explored γ-secretase inhibitors as a way to block Notch signaling and potentially sensitize tumors to chemotherapy.^416,417^ Early clinical trials have also explored inhibitors of the Hedgehog signaling pathway, which modulates CSC self-renewal and drug resistance, but efficacy has been context-dependent and translational benefit remains limited. Beyond targeting CSC-specific pathways and their microenvironment, CSC-directed immunotherapies, including cancer vaccines, checkpoint inhibitors, and chimeric antigen receptor T-cell (CAR-T cell) therapies, have opened new therapeutic possibilities.^418^ Despite these advancements, there remains a critical need for innovative strategies that accurately target CSCs while sparing normal stem cells, given the complexity of CSC biology and the intricate interactions of their signaling pathways.^165^

## Emerging therapies and approaches

The landscape of cancer treatment is being transformed by the introduction of innovative therapies and methods that primarily target CSCs. In recent years, several multidimensional approaches have been utilized to target specific markers and pathways to eliminate CSCs, alter the tumor microenvironment, induce differentiation or apoptosis, resensitize tumors to chemotherapy, and reverse EMT. These advancements aim to address the specific challenges associated with CSCs, thereby enhancing the overall effectiveness of cancer treatments by preventing tumor relapse and spread.

### Novel therapeutic agents and strategies

In recent years, there has been a significant advancement in the development of targeted inhibitors focused on signaling pathways critical to the survival and proliferation of CSCs. A prominent approach involves targeting the WNT/β-catenin pathway, which is known for maintaining the proliferation and stemness properties of CSCs.^12,39^ There has been progress in developing Porcupine inhibitors that impede the secretion and activity of WNT ligands, thereby interrupting the WNT/β-catenin signaling pathway.^419^ These inhibitors have shown encouraging outcomes in preclinical studies, highlighting their potential as effective treatments against CSCs. Moreover, immunotherapy has emerged as a revolutionary strategy in cancer treatment, with ongoing refinements to specifically target CSCs. This includes the development of vaccines designed to initiate an immune response against CSC-specific antigens and the use of checkpoint inhibitors that may indirectly affect CSC-enriched niches by restoring antitumor immunity and support immune-mediated tumor-cell elimination.^420,421^ In particular, immune checkpoint inhibitors targeting CTLA-4 and PD-1/PD-L1 have demonstrated efficacy in several cancers although CSC-specific activity requires dedicated biomarkers and functional validation.^421–424^ Such immunotherapeutic approaches offer a promising avenue for directly combating CSCs, thereby potentially reducing the likelihood of tumor recurrence and metastasis. However, CSC-specific clinical activity should be interpreted cautiously unless CSC-directed biomarkers or endpoints were measured.

Additionally, inflammation that promotes tumorigenesis has been implicated in the initial stages of cancer progression and in the development of therapeutic resistance. While the mechanisms by which repurposed agents and natural products target CSCs remain to be fully elucidated, compounds such as salinomycin, curcumin, and metformin are being investigated for CSC effects, including ferroptosis induction, autophagy-linked suppression of CSCs, and reduced cancer stemness-associated signaling.^425–427^

### Combination therapies and multimodal approaches

In addressing the challenge posed by the resilience and therapeutic resistance of CSCs, combination therapies and multimodal strategies have been investigated. The core principle of combination therapy lies in targeting multiple pathways or facets of CSC biology simultaneously, including their interactions within the tumor microenvironment.^428^ This approach aims to curtail the CSCs’ capacity for adaptation and resistance, potentially enhancing the effectiveness of cancer treatments. The rationale behind using combination therapies is to dismantle the complex networks that support CSCs, thereby significantly impeding their functionality and improving treatment outcomes. Several clinical trials evaluated the efficacy of integrating CSC-relevant pathway inhibitors with traditional chemotherapy, but outcomes have been mixed and often limited by toxicity, lack of biomarker selection, and absence of direct CSC endpoints.^429–431^

These emerging therapies and approaches represent a paradigm shift towards more targeted and potentially more effective cancer treatment regimens. By focusing on CSCs, which are often at the heart of treatment resistance, tumor recurrence, and metastasis, these innovative strategies hold the promise of longer-lasting responses and improved survival rates for cancer patients.

## Clinical perspectives and future directions

A major clinical challenge in targeting CSCs is their multifactorial and context-dependent response to therapy. CSCs rely on malignant signaling networks and interactions with the tumor microenvironment, show marked heterogeneity and metabolic flexibility, and lack widely accepted biomarkers that translate across tumor types.^10^ These factors contribute to CSC-associated drug resistance, making single-agent approaches limited to deliver durable clinical benefit. As a result, combination regimens may be needed to increase antitumor activity and improve overall survival (OS). Recent CSC-oriented clinical trials have shown limited improvements in patient outcomes. Table 2 summarizes leading clinical attempts to date.

**Table 2:** Summary of clinical studies that directly target CSC-associated antigens or indirectly target CSC-related pathways, niches, or stemness programs across tumor types

Agent/intervention	Target/pathway relevant to CSCs	Indication	Trial phase/status	Key findings	Major challenges	Reference/clinical trial ID
Ipafricept (OMP-54F28)	WNT ligand decoy (FZD/WNT)	Ovarian cancer/Pancreatic adenocarcinoma	Phase 1b	Published phase Ib studies reported tolerability and limited clinical activity in ovarian cancer and pancreatic adenocarcinoma combinations; bone-related toxicity and program-level development constraints limited further translation.	Single-agent efficacy remains unproven. Bone safety, biomarker-guided stratification, optimized dosing and pathway redundancy remain major barriers.	NCT02092363, NCT02050178, ^429,430^
Napabucasin (BBI-608)	Cancer stemness through suppression of STAT3	Several solid tumors including pancreatic, gastric, colorectal, and other tumors	Multiple phases	Minor anti-tumor response. Controlled disease course. Larger trials reported either mixed or negative results for primary endpoints.	Heterogeneous patient selection, absence of validated stemness biomarkers, and insufficient efficacy in larger trials limited translation.	^431,489^, NCT02753127
CD133-directed CAR-T (CART-133)	CD133 (PROM1) surface antigen on CSC-enriched tumor cells	Advanced CD133-positive metastatic malignancies, including hepatocellular, pancreatic, colorectal, and other solid tumors	Phase I	Feasible with manageable toxicities. Anti-tumor responses and disease control were reported in subsets.	Heterogeneous CD133 expression, on-target/off-tumor toxicity risk, limited persistence, and immunosuppressive TME barriers.	^437^
Autologous CD133 dendritic-cell vaccine (ICT-121)	CD133 peptides presented by autologous dendritic cells; CSC-associated antigen vaccination	Recurrent glioblastoma	Multi-center phase I translational/clinical study	Safety and tolerability were reported, and immune responses were observed in a subset of patients.	Early-phase study with a small cohort; efficacy, durability, and biomarker correlations require randomized validation.	^438^
Disulfiram + Cu (repurposed ALDH inhibitor)	ALDH family - metabolic stemness marker.	Multiple solid cancer trials combining with chemo/radiation.	Multiple Phase I/II trials	Acceptable safety profile. Some anti-tumor response reported. Feasible biomarkers in some studies.	No clear clinical outcomes. Beneficial only to some subsets of the cohort. Reported toxicities.	NCT01907165, NCT00742911, ^432,433^
Bivalent CAR-T combination (EGFR806/IL13Rα2)	Tumor-associated EGFR/IL13Rα2 antigens; not CSC-specific markers	Recurrent EGFR-amplified glioblastoma	Phase I; active/not recruiting in registry; published interim data available	Published phase I data reported feasibility and preliminary radiographic/biological activity in selected patients; mature registry-posted results and long-term efficacy data remain limited.	Neurotoxicity and inflammatory toxicity require careful monitoring; dose refinement, patient selection, tumor heterogeneity and persistence of infused cells remain challenges.	NCT05168423, ^435^
Vismodegib	Hedgehog pathway inhibitor (SMO)	Advanced periocular basal cell carcinoma	Phase II	Tumor regression and preservation of visual function were reported in a disease-specific BCC setting.	Resistance can emerge through SMO mutations or pathway bypass; biomarker-guided selection and management of adverse effects remain important.	NCT02436408, ^490^
Metformin	OXPHOS/metabolic modulator	Ovarian cancer	Phase II	Well tolerated. Direct metabolic biomarkers. Reasonable clinical outcomes evaluation.	Single-arm design without randomized control and heterogeneity of CSC metabolism limit interpretation.	NCT01579812, ^491^
Doxycycline	Mitochondrial biogenesis inhibitor	Pancreatic cancer	Phase II	Translational potential for biomarker testing. Already characterized drug with repurposing potential.	No clinical outcomes defined. Only pathological endpoints. Small cohort.	NCT02775695
Defactinib (VS-6063)	FAK inhibitor, (targets CSC niche/adhesion signaling)	NSCLC (KRAS mutant) and other solid tumors	Phase II	Partial clinical outcomes. Acceptable tolerability and safety profile.	Modest activity as a single agent. Poor correlation between biomarkers and clinical efficacy.	NCT01951690
Reparixin	CXCR1 inhibitor (regulates IL-8 axis on CSCs).	Triple-negative breast cancer (TNBC)/HER2-neg metastatic breast cancer.	Phase I/Phase II	Basis for proof-of-concept with safety and tolerance profile established. Biomarker outputs suggest that it can regulate CSC.	No clinical benefit in a larger randomized phase relevant to CSC targeting. Translational gap: No clear clinical benefit compared to preclinical CSC suppression.	NCT02370238, NCT02001974
MK0752	γ-secretase inhibitor (Notch pathway inhibitor).	Advanced/metastatic breast cancer.	Phase I/II	In dose-escalation testing, the safety of combination therapy was manageable and preliminary clinical activity was reported in a subset.	Reliable biomarker selection and direct validation of CSC targeting are needed before clinical benefit can be attributed to Notch-pathway inhibition.	NCT00645333
IPI-926 (Saridegib)	Hedgehog pathway inhibitor	Head & neck cancer (and other solid tumors)	Phase I/Ib	Combinatorial effect. Acceptable safety.	Limited clinical translation. Off-target effects and toxicities.	NCT01255800, ^492^
Fursultiamine	Inhibits ABCB1/ABCG2 transporter (Reverse drug efflux in CSCs)	Esophageal squamous cell carcinoma	Phase II	An established safety profile made repurposing feasible. The addition of the CSC biomarker to the endpoints highlights the potential of follow-up.	Single-arm design without randomized control. The CSC mechanism is less clinically characterized than established pathway targets, and published efficacy data are limited.	NCT02423811
Imetelstat	Telomerase inhibitor	NSCLC	Phase II	Mechanistically interesting approach targeting a CSC maintenance mechanism aimed at reducing minimal residual disease after therapy.	No improvement in clinical outcomes was reported, reflecting limited translational potential in this setting. Biomarker validation remains unresolved, including whether telomere-related markers predict outcomes.	NCT01137968
CSC vaccines	CSC-associated or CSC-enriched antigen-based vaccines	Nasopharyngeal, hepatocellular, lung, ovarian, colorectal, pancreatic; TNBC.	Multiple early-phase trials.	Clinical efficacy has generally been modest, although acceptable safety and some immune or antitumor responses have been reported in early-phase settings.	Improved antigen validation, patient selection and combination with immune-checkpoint or TME-modulating approaches may be needed because CSC-associated antigens are heterogeneous.	NCT02115958, NCT02089919, NCT02084823, NCT02178670, NCT02176746, NCT02074046, NCT05455658
DC vaccine	Targeting CSC antigens	Glioblastoma	Phase II/III (recruiting)	The DEN-STEM vaccine is designed to target CSC-associated antigens. No mature efficacy results have been published.	To demonstrate meaningful efficacy, future analyses would ideally show CSC-burden reduction that correlates with clinical outcomes.	NCT03548571
CSC antigen-targeted vaccine	Vaccine derived from CSC antigen lysate	Glioblastoma	Phase I (completed)	The phase I study demonstrated feasibility and safety.	Efficacy remains unclear and requires larger randomized cohort studies with CSC-relevant immune and clinical endpoints.	NCT02010606, ^493^
Vismodegib with Gemcitabine	Hedgehog inhibitor with chemotherapy	Pancreatic cancer	Phase Ib/II	Phase Ib/II. The combination explored Hedgehog inhibition with chemotherapy in metastatic pancreatic cancer, but the randomized study did not show significant improvement in ORR, PFS, or OS compared with control.	No validated biomarker identified a responsive CSC-dependent subgroup. Limited clinical benefit suggests pathway redundancy, insufficient CSC targeting, or context-dependent stromal effects.	NCT01195415, ^494^
ChemoID-guided therapy	Ex vivo-guided chemotherapy against CSC (ChemoID)	Recurrent glioblastoma	Phase III	An interim randomized analysis suggested possible improvement in clinical outcomes and illustrates a personalized ex vivo-guided therapy approach.	Results are from interim analysis and may need longer follow-up to draw definitive conclusions. Logistics including sampling, culturing as well as assay reproducibility/standardization. Validation required due to limited chemotherapeutic options.	NCT03632135, ^495^
Bevacizumab	Targeting the CSC TME.	Breast cancer	Phase II	Translational potential could not be established. The study may indicate that anti-angiogenic monotherapy is insufficient to remodel the CSC-supportive TME.	Limited clinical outcomes might be related to TME suppressor effect or CSC resistance. Limited biomarker selection. CSCs are highly heterogeneous.	NCT01190345
Temsirolimus with Liposomal Doxorubicin	mTOR inhibitor with chemotherapy	Sarcoma	Phase I/II	Controlled disease. Feasible safety profile. Signals clinical activity were reported. Biomarker was correlated with the response.	Single-arm design limits attribution of benefit to CSC-related biology; the biomarker remains a surrogate requiring validation.	NCT00949325, ^496^

### Targeting CSC biomarkers

Several clinical trials have attempted to target CSC-associated markers across tumor types, with limited success. Common reasons include suboptimal biomarker selection, CSC plasticity and pathway redundancy, and challenges in drug delivery and intratumor exposure. For example, multiple trials evaluated disulfiram/copper in glioblastoma, partly motivated by ALDH-associated stemness biology.^432–434^ However, these studies do not establish ALDH-positive CSC depletion as the clinical mechanism. Results were largely inconclusive or negative, attributed in part to limited intratumor penetration and off-target effects that increased toxicity. Despite supportive preclinical evidence, these outcomes suggest limits of current models for predicting clinical activity in glioblastoma patients.

### CSC-directed immunotherapy

Many immunotherapy strategies have entered clinical use, including CAR-T therapy in selected hematologic malignancies. Solid tumors remain difficult due to a complex microenvironment, CSC plasticity, and treatment-related toxicities. These factors can sustain an immunosuppressive milieu that supports immune evasion and resistance, while many target antigens are shared with normal tissues, creating on-target, and off-tumor toxicity risks. Only a small number of clinical programs have explicitly focused on CSC-directed immunotherapy. Multi-antigen CAR-T designs may help address tumor heterogeneity and antigen escape. Tandem CARs (TanCARs) that recognize EGFRvIII and IL13RA2, and bivalent CARs engineered to target these antigens, have shown encouraging activity in preclinical work and early clinical studies in glioblastoma.^435,436^ Clinical trial experience also supports feasibility and manageable toxicity for CAR-T therapy directed against CD133 in advanced malignancies.^437^ In addition, autologous CD133 dendritic cell immunotherapy (ICT-121) demonstrated safety and tolerability, with immune responses observed in a subset of recurrent glioblastoma patients.^438^

Other immunotherapies are designed to redirect T-cells and Fc-gamma receptor I/III to the tumor cells. Catumaxomab, a trifunctional antibody directed against EpCAM and CD3, was tested intraperitoneally in EpCAM-positive ovarian cancer patients with malignant ascites in a phase I/II dose-escalation study.^439^ Catumaxomab reduced malignant ascites accumulation with an acceptable safety profile. Interpretation is limited by cohort size and the absence of a control arm, and longer follow-up is needed to define durability and delayed adverse effects.

Mechanistic studies have connected LGR5 binding to selective EGFR degradation in LGR5-positive cancer cells through petosemtamab, a bispecific antibody that binds LGR5 and can promote EGFR degradation.^40,440,441^ Phase I/II trials reported clinical responses in refractory or metastatic head and neck squamous cell carcinoma (HNSCC), consistent with a role for LGR5-positive CSC-like cells in resistance in this population. Ongoing randomized phase III trials (LiGeR-HN1 and LiGeR-HN2; NCT06525220 and NCT06496178) are evaluating petosemtamab alone or with pembrolizumab in recurrent/metastatic HNSCC.^441^

In metastatic castration-resistant prostate cancer, a phase I trial targeted prostate stem cell antigen (PSCA) with CAR-T cells and reported an acceptable safety profile with signals of clinical activity and informative correlative data for biomarker development and combination hypotheses.^442^ However, durable remissions in solid tumors may require longer follow-up and strategies to extend persistence and function of infused cells, since sustained responses beyond 28 days post-infusion were limited. Defining the mechanisms behind short response duration may inform improved trial design, patient selection, and combination strategies.

### CSC-targeted vaccines

Vaccine approaches aimed at CSC-associated antigens include dendritic cell (DC)-based vaccines, peptide vaccines, and mRNA-based platforms. The shared goal is to induce immune responses that suppress residual disease and reduce recurrence. In an early study, a pancreatic CSC-derived vaccine showed dose-associated post-vaccination immune responses and a tolerable safety profile, but did not report clear clinical benefit for recurrence-free survival or OS.^443^ Limited long-term follow-up also leaves uncertainty about whether the observed immune response provided durable protection.

In newly diagnosed patients with glioblastoma, promising results from phase II trial were achieved using an autologous DC vaccine against six antigens on both tumor and CSCs (ICT-107). Progression free survival (PFS) was significantly improved in ICT-107-treated patients, and patients in one of the targeted antigens, HLA-A2 subgroup, demonstrated increased clinical and immunological ICT-107 activity.^444^ Another phase I/II study demonstrated excellent safety and tolerability profile and a potent and long-lasting T-cell immunity among prostate cancer (PCa) patients receiving peptide-based vaccination targeting RhoC. RhoC is a small GTPase overexpressed in many advanced solid cancers, metastases, and CSCs.^445^ The results of this trial have encouraged to examine the efficacy of the vaccine in a double-blind, placebo-controlled, phase II trial for PCa patients with biochemical recurrence following curatively intended therapy for localized prostate cancer (NCT04114825). Although the vaccine continues to show encouraging safety and tolerability, it failed to meet its primary clinical efficacy goal (Prostate-specific antigen (PSA) doubling/progression). It is not clear whether delaying PSA progression could be translated into clinically meaningful benefit such as PFS or OS. Moreover, there was no direct correlation between the immune response elicited by the vaccine and its clinical efficacy, for instance, delaying the PSA progression in this case. Indeed, T-cell activation cannot always be translated into tumor control in practice which often seen in cancer vaccine trials.

Another promising avenue is the use of mRNA vaccines. One previous clinical trial used DC-based vaccine targeting CSCs in glioblastoma patients demonstrated immune response induced by the vaccination which was well tolerated by patients and prolonged PFS.^446^ The safety profile of the vaccination and the preliminary results have led to move to a randomized phase II/III clinical trial aimed at employing dendritic cells transfected with mRNA of survivin and telomerase reverse transcriptase (hTERT) from autologous CSCs in similar group of patients receiving standard therapy (DEN-STEM, NCT03548571). Another ongoing study (STEMVAC) is using multiple antigens associated with breast CSCs including Endoglin (CD-105), YB-1, SOX2, Cadherin 3 (CDH3), and MDM2 in phase II against TNBC in multiple trials (NCT05455658, NCT07078604, NCT07112053). While few ongoing clinical studies are in their early phases, mostly exploring the safety profile of the vaccinations, more studies are still needed prior to any firm conclusions regarding the clinical efficacy of the CSC-based vaccines.

### Targeting the CSC microenvironment

The TME plays a crucial role in providing CSCs with essential cues required to survive. CSCs are usually surrounded by structural levels of complex cellular cross-talks and signaling networks, orchestrating together massive regulatory intrinsic and extrinsic processes which make targeting CSCs challenging. Preclinical and clinical evidence supports the TME as a therapeutic target in cancer and CSC biology, but direct CSC-specific clinical evidence remains limited.^447–450^ Examples such as pexidartinib/durvalumab and mogamulizumab/nivolumab illustrate the complexity of modulating immune niches rather than direct CSC eradication.^451–453^ While the trial demonstrated tolerable safety profile, the synergy could not achieve clinical efficacy. In fact, one reason explaining failure to produce an immunological response is the off-target effect of the drug on DCs mediated through FLT3 inhibition both of which are essential in normal hematopoiesis and in initiation of anti-tumor T-cell response. It is worth mentioning here that both colorectal and pancreatic cancers targeted in the study are characterized by low mutational burden and lower baseline T-cell levels. Therefore, any negative effect on immune response-mediating cells like DCs may have a substantial negative impact on the drug synergy. Nevertheless, the study highlights the complexity of targeting the TME in which targeting one cell subset could impact the others. Another clinical attempt to target CSC immunosuppressive niche was through regulating Tregs in the TME by targeting CCR4 and PD-1 in patients with solid tumors (NCT02946671).^454^ Although this early trial phase achieved its safety objective, it remains early to draw solid conclusions about tumor type response to the combination due to the small number of patients and heterogeneous tumor cohort. Moreover, an ongoing clinical trial using CXCR1/2 inhibitor (SX-682) in patients with metastatic melanoma undergoing treatment with anti-PD-1 is assessing the potential of targeting myeloid-derived suppressor cells (MDSCs) demonstrated initial increased cytotoxic T-cell response and reduced MDSCs infiltration (NCT03161431). While these clinical data remain preliminary, they provide initial evidence of anti-tumor activity by harnessing the TME and provide a basis for larger clinical trials.

Other clinical trials that indirectly targeted CSC-mediated pathways did not measure CSC burden as a direct endpoint, limiting interpretation of CSC-specific effects. For example, a dose-escalation study of CB-103, a small-molecule inhibitor of the CSL-NICD interaction in advanced solid tumors and hematologic malignancies, suggested that CB-103 monotherapy may not be sufficient to achieve meaningful clinical outcomes.^455^ Even et al. investigated the safety and antitumor activity of crenigacestat (LY3039478), a selective oral Notch inhibitor, in an expansion cohort of patients with metastatic adenoid cystic carcinoma (ACC). Although disease stability was reported, gastrointestinal toxicity was a major adverse effect, and there were no direct CSC endpoints because the trial focused on conventional outcomes such as PFS/OS rather than CSC fraction.^456^ Another trial, based on promising preclinical studies, concluded that vismodegib plus gemcitabine and nab-paclitaxel in untreated metastatic pancreatic cancer did not reduce CSC burden, alter stromal components, or improve survival.^457^ In patients with basal-cell nevus syndrome, vismodegib reduced basal-cell carcinoma (BCC) tumor burden and growth in a randomized clinical trial. Although this did not directly prove CSC targeting, relapse after discontinuation could involve persistence of drug-tolerant cells with stem-like characteristics.^458^

On the other hand, a study evaluated the efficacy of nirogacestat, a γ-secretase inhibitor, in adults with progressing desmoid tumors demonstrated significant PFS improvement with a 41% objective response rate and low-grade reported adverse events.^459^ Although this potential effect may be relevant to NOTCH/γ-secretase inhibition, it is worth mentioning that desmoid tumors are not typically considered classical CSC-driven tumors. This might indicate that the clinical activity course of some tumors could be biologically different from stem cell-driven high-grade solid tumors.

While these data did not measure CSC endpoints or stratified patients by CSC biomarkers and are mostly pathway-targeted, and in some cases cancer type-dependent, it suggested a possible clinical relevance of CSC-related pathways and support further evaluation of targeting CSCs.

### Future directions and conclusions

Future clinical progress will likely depend on three connected steps: biomarker-guided patient selection, trial endpoints that capture CSC effects, and rational combination strategies that address both CSC-intrinsic programs and the TME.

Reliable CSC biomarkers remain a central need for identifying patients most likely to benefit from CSC-targeted interventions.^70^ Future biomarker development should distinguish traditional surface-marker panels from functional-state biomarkers. Surface markers such as CD44, CD24, CD133, EpCAM, LGR5, and CD166 are useful for enriching or gating CSC-like populations. However, they mainly identify phenotypic fractions, not functional states. These markers can be broadly expressed by normal tissues, vary by isoform and threshold, and may change after dissociation, sorting, therapy or niche perturbation.^7,10,40,51,52,70,92–94^ As a result, surface-marker positivity alone does not necessarily indicate whether a tumor cell is quiescent, EMT-primed, hypoxic, OXPHOS-dependent, ferroptosis-resistant, or therapy-tolerant at the time of sampling.^10,48,59,240,348^

By contrast, functional-state biomarkers report active biological programs or vulnerabilities, rather than lineage identity alone. Examples discussed in previous sections include ALDH activity, ABC-transporter dye efflux, ROS tone, HIF target activity, WNT/Notch/Hedgehog reporter activity, OXPHOS and fatty-acid-oxidation programs, mitochondrial fusion or mitophagy status, and single-cell or spatial signatures of EMT, dormancy, and niche dependence. Because these readouts are dynamic, they may require longitudinal sampling, single-cell or spatial profiling, protein–RNA co-measurement, and ex vivo functional testing.^103,106,112,460^ Even so, they may better align patients with therapies that target plasticity, metabolism, or stress-adapted residual disease. Standardized assay workflows and cross-platform validation will be important for reproducible use in multicenter trials.

Trial design should also evolve to measure CSC modulation as a defined endpoint rather than relying only on bulk tumor response. Serial sampling through liquid biopsy, circulating tumor DNA, circulating tumor cell profiling, or tumor biopsies when feasible can be combined with functional assays to track CSC burden or state over time. Embedding these measures into adaptive trials may allow treatment adjustments based on early biomarker shifts, rather than waiting for radiographic progression. Patient-derived organoids and related ex vivo platforms can support personalized functional testing and combination prioritization,^58,461–463^ but they should be benchmarked prospectively against clinical outcomes and CSC-aware endpoints before being treated as validated CSC-guided assays.

Combination strategies should be built from mechanism-based hypotheses and validated with pharmacodynamic readouts. Pairing pathway inhibitors that affect CSC maintenance programs, for example Notch, WNT or Hedgehog signaling, with immune checkpoint blockade, anti-angiogenic agents, or TME-modulating therapies may disrupt CSC support while improving immune-mediated control. Metabolic targeting of glycolysis or oxidative phosphorylation may also sensitize CSC-like populations to chemotherapy or radiotherapy, depending on tumor context. Co-targeting hypoxia pathways, cancer-associated fibroblast programs, or myeloid immunosuppression may further weaken CSC persistence, but safety and immune competence must be monitored closely, given the risk of unintended suppression of antigen presentation or effector-cell function.

Drug delivery remains another practical barrier. Nanoparticle-based delivery, intratumoral or regional administration where appropriate, and approaches that improve tumor penetration may reduce systemic toxicity while improving exposure in CSC-rich niches. Genome editing tools such as CRISPR-based systems may enable target validation and, in selected settings, therapeutic strategies, but clinical translation will require careful safety engineering and delivery solutions. Integrating multi-omics with machine learning may improve prediction of resistance programs and prioritize combination regimens, especially when linked to longitudinal biomarker data collected during trials.

Taken together, progress in CSC-directed therapy will likely come from trials that connect mechanistic biomarkers, CSC-aware endpoints, and rational combinations, while maintaining close attention to toxicity and drug delivery. Integrating standard therapies, immunotherapy, and CSC-focused approaches in biomarker-guided frameworks may reduce relapse and improve durability of response in selected patient groups.

## Data Availability

No new datasets were generated or analyzed in this review. All data discussed are available in the cited literature, public clinical-trial registries, and associated public resources.
